# The quality of reporting in randomized controlled trials of acupuncture for knee osteoarthritis: A cross-sectional survey

**DOI:** 10.1371/journal.pone.0195652

**Published:** 2018-04-12

**Authors:** Pengli Jia, Li Tang, Jiajie Yu, Jiali Liu, Deying Kang, Xin Sun

**Affiliations:** Chinese Evidence-based Medicine Center, West China Hospital, Sichuan University, Chengdu, Sichuan, China; Stanford University School of Medicine, UNITED STATES

## Abstract

**Objective:**

To assess the reporting quality of acupuncture trials for knee osteoarthritis (KOA), and explore the factors associated with the reporting.

**Method:**

Three English and four Chinese databases were searched from inception to December 2016 for randomized control trials testing effects of acupuncture for knee osteoarthritis. We used the standard CONSORT (2010 version), CONSORT Extension for Non-Pharmacological Treatments, and STRICTA for measuring the quality of reporting. Using pre-specified study characteristics, we undertook regression analyses to examine factors associated with the reporting quality.

**Results:**

A total of 318 RCT reports were included. For the standard CONSORT, ten items were substantially under-reported (reported in less than 5% of RCTs), including specification of important changes to methods after trial commencement (0.6%), description of any changes to trial outcomes (0.0%), implementation of interim analyses and stopping guidelines (0.6%), statement about why the trial ended or was stopped (1.6%), statement about the registration status (4.4%), accessibility of full trial protocol (4.7%), implementation of randomization (4.7%), description of the similarity of interventions (3.5%), conduct of ancillary analyses (3.8%) and presentation of methods for additional analyses (4.4%). Four of the STRICTA items were under-reported (reported in less than 10% of RCTs), including description of acupuncture style (8.5%), presentation of extent to which treatment varied (1.3%), statement of practitioner background (7.2%) and rationale for the control (9.1%). For CONSORT Extension, the reporting was poor across all items (reported in less than 10% of trials). Trials including authors with expertise in epidemiology or statistics, published in English, or enrolling patients from multiple centers were more likely to have better reporting.

**Conclusions:**

The reporting in RCTs of acupuncture for KOA was generally poor. To improve the reporting quality, journals should encourage strict adherence to the reporting guidelines.

## Background

Randomized controlled trials (RCTs) are the gold standard for assessing the effects of health care interventions [1]. However, RCTs may yield misleading results if they lack methodological rigors [2]. Adequate reporting of RCTs is one of critical methodological issues, since the information reported has profound impact on the decisions by healthcare professionals and policy makers. Previous studies showed that RCTs with poor reporting, compared to those with good reporting, yielded larger effect estimates across a variety of healthcare conditions [3].

In order to improve the reporting of RCTs, scientific communities have made great efforts to develop recommendations, such as the Consolidated Standards of Reporting Trials (CONSORT) statement which aims to improve the general reporting of RCTs [4, 5]; the CONSORT extension for nonpharmacological treatments which addresses the reporting issues specific to complex interventions, such as surgery, devices, rehabilitation, psychotherapy, behavioral interventions, and complementary and alternative medicine [6]; and the Standards for Reporting Interventions in Clinical Trials of Acupuncture (STRICTA), a recommendation for the descriptions of acupuncture treatments [7].

Acupuncture is an important healthcare intervention. Recent years have seen burgeoning increase of RCTs testing effects of acupuncture. Compared to drug trials, acupuncture interventions are typically complex, patient recruitment is often difficult, and standardization of intervention is more challenging. Consequently, reporting of acupuncture trials is more complex, and careful and meticulous reporting is of paramount importance. Several studies have examined the issue of reporting among acupuncture trials, and identified a number of issues regarding inadequate reporting [8–12].

Nevertheless, none specifically examined the reporting of RCTs testing acupuncture knee osteoarthritis (KOA) [13]. As a traditional intervention, acupuncture has been widely used to treat osteoarthritis disorders in China as well as developed countries [14, 15]. In the US, about one million patients used acupuncture to treat musculoskeletal disorders [16]; between 30% and 40% of general practices in England provide complementary treatment options for patients with KOA, among which acupuncture is the most popular choice [17]. Lack of adequate reporting of details in such trials would make the effective use of trial evidence less likely. Even in certain circumstances, this would lead to misled healthcare decisions. Therefore, we conducted a cross-sectional survey to specifically assess the extent to which the current RCTs examining acupuncture for KOA comply with the recommendations by the established reporting standards, and explore factors associated with the reporting.

## Methods

### Study selection

We included RCTs published either in English or Chinese as full-text reports that enrolled patients diagnosed with KOA, and compared acupuncture versus a control. We defined acupuncture as a stimulation of the body or auricular points regardless of the type of stimulation [9]. Any type of acupuncture was eligible for inclusion, such as electro-acupuncture, filiform needle, fire needle, silver needle, dry needle, laser acupuncture, ear acupuncture, and scalp acupuncture, regardless of the duration of treatment. The control may include acupuncture, pharmacologic intervention, placebo acupuncture (placing needle on the surface of skin without penetration), sham acupuncture (placing needle on sham points near acupuncture point), waiting list, and physical treatments (e.g. exercises, weight loss). RCTs that combined acupuncture with moxibustion were eligible, if using moxibustion as a co-intervention across groups.

### Data sources

We searched PubMed, EMBASE and Cochrane Central Register of Controlled Trials (CENTRAL) and four Chinese Databases, including Chinese Biomedical Database (CBM), National Knowledge Infrastructure(CNKI), Wanfang and VIP, all from the inception to December 2016. The search terms were customized for each individual databases (S1 File). Reference lists of all eligible trial reports were screened for additionally eligible studies.

### Study process

Two investigators (PLJ and JLL) independently screened titles and abstracts for potential eligibility. They subsequently read full texts of potentially eligible reports for final eligibility. Then, the two investigators (PLJ and JLL) independently assessed the quality of reporting of the eligible RCT reports. Any disagreements were resolved through discussion.

### Data collection

We collected the information regarding study characteristics from each eligible RCT, as follow: name of first author, year of publication, journal name, journal type, sample size, number of groups, length of follow up, funding source (not-for-profit funding, for profit funding, clearly stated, not funded and not reported) and statistical significance of the primary outcome (p<0.05). When there was no clearly specified primary outcome or there was more than one primary outcome, we used the pre-specified criteria for selecting a primary outcome (S2 File) [18].

In order to measure the quality of reporting, we used the CONSORT Statement (2010 version) and the CONSORT Extension for Nonpharmacologic Treatments. The standard CONSORT recommendations contain 36 items [5], and the CONSORT extension include 13 additional items [6]. We also used STRICTA to measure details specific to acupuncture (17 items) [7]. This resulted in a total of 63 items for the finial questionnaire. Each item was assigned one of the three response options: ‘Yes’ for compliance of reporting, ‘No’ for noncompliance, and ‘NA’ representing that the item was not applicable (S1 Table).

We developed data forms according to the checklists, and pilot-tested the forms by one author (PLJ). Then, a group discussion was undertaken to clarify the definition of each item. Thereafter, the assessment was calibrated using a random sample of 15 reports. Finally, data extraction was performed by two investigators (PLJ and JLL).

### Data analysis

Descriptive statistics were used to summarize the characteristics of included studies. Dichotomous data were presented as number and percentage and continuous variables were described as median with interquartile range (IQR).

We summarized the score according to the checklist. Each item was given one point if reported by a study, otherwise zero point was given. The maximum possible scores for the standard CONSORT checklist, CONSORT extension, and STRICTA were 36, 10 and 17 points. We calculated the total score by adding each of the component checklists (i.e. standard CONSORT plus CONSORT extension and STRICTA) and the score for standard CONSORT (i.e. CONSORT score).

To examine the association of reporting quality with study characteristics, we pre-specified five factors. We initially listed potentially relevant factors based on our hypotheses and findings from previous reports. Then, the study team, consisting of clinical trial experts, statisticians and acupuncturists, discussed their relevance to our study. Our discussion ended up with five factors, including author’s affiliations to the epidemiology or statistics department (yes vs no) based on the information included in the RCT reports, language (English vs Chinese), multi-center study (yes vs no), sample size (sample size ≤80 vs > 80, categorized according to the median) and significance of primary outcome (P < 0.05) (yes vs no).

We used univariable and multivariable linear regression analyses to examine the association of reporting quality with the pre-specified variables. We conducted two set of analyses, one for the overall score (i.e. standard CONSORT, CONSORT extension plus STRICTA), and one for the standard CONSORT score. We checked and assured that the scores did not appear to violate the assumption of normality.

In order to examine the impact of the scoring approach on the regression analysis, we conducted one sensitivity analysis, in which we assigned one point to an item if it was reported by the trial under assessment or not applicable. We then explored the association between the putative factors with the generated scores.

## Results

### Study searching and selection

**Fig 1:**
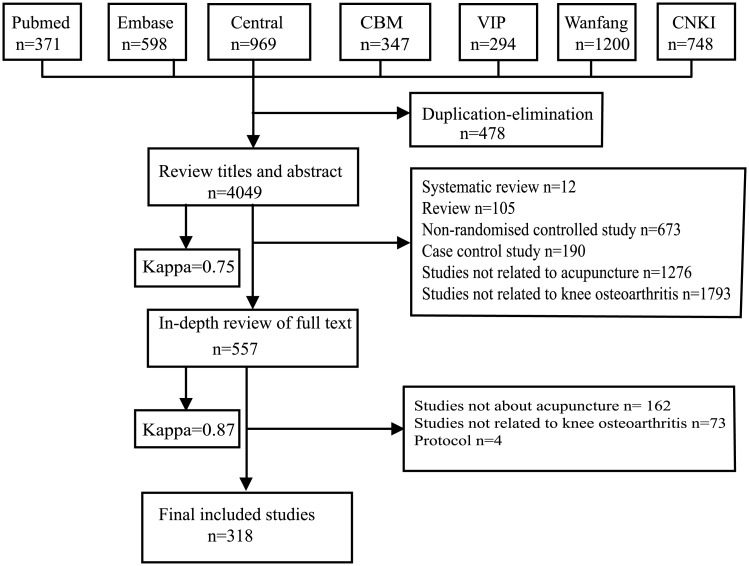
Flow diagram for searching and selection processes.

The search yielded 4,527 reports. After title and abstract screening, 557 reports were potentially eligible; upon reading full texts, 318 RCT reports proved eligible (Fig 1). The details of included RCTs were listed in S3 File.

These 318 RCTs were published between 1992 and 2016, among which 264 (83.0%) were published in Chinese, 267 (84.0%) were two-arm trials, 278 (87.4%) were single-center trials, and 98 (30.8%) were funded by not-for-profit funding agencies (Table 1). Eight (2.5%) RCTs were conducted in the UK [17, 19–25], ten (3.1%) in the USA [26–37], and six (1.9%) in Germany [19, 38–42]. The sample sizes ranged from 12 to 1039 (median: 80). The length of follow up ranged from 1 to 48 weeks (median: 3 weeks). Only 31 (9.7%) trials were registered and 22 (6.9%) mentioned the study protocol. The median number of authors was 3 (interquartile range 2–5).

**Table 1:** General characteristics of included RCTs.

Features of included RCTs	Total (n = 318, %)	Chinese (n = 264, %)	English (n = 54, %)
**Sample size [Median (IQR)]**	76 (60–100)	80 (60–105)	60 (40–92)
≤80^b^	167 (52.5)	135 (51.1)	32 (59.3)
>80	151 (47.5)	129 (48.9)	22 (40.7)
**Involving research centers**			
Single center	278 (87.4)	245 (92.8)	33 (61.1)
Multi-center	40 (12.6)	19 (7.2)	21 (38.9)
**Number of arms**			
2 arms	267 (84.0)	234 (88.6)	33 (61.1)
>2 arms	51 (16.0)	30 (11.4)	21 (38.9)
**Length of follow up[Median (IQR)]**	2 (1–7)	2 (2–5)	3 (1–8)
≤3 weeks^b^	113 (35.5)	103 (39.0)	10 (18.5)
>3 weeks	205 (64.5)	161 (61.0)	44 (81.5)
**Sources of trial funding**			
Not-for-profit funding	98 (30.8)	64 (24.2)	34 (62.0)
For profit funding	0 (0.0)	0 (0.0)	0 (0.0)
Clearly stated, not funded	0 (0.0)	0 (0.0)	0 (0.0)
Not reported	220 (69.2)	200 (75.8)	20 (37.0)
**Whether a protocol was explicitly mentioned**			
Yes	22 (6.9)	0 (0.0)	22 (40.7)
No	296 (93.1)	264 (100.0)	32 (59.3)
**Whether the study was registered**			
Yes	31 (9.7)	0 (0.0)	31 (57.4)
No	287 (90.3)	264 (100.0)	23 (42.6)
**Number of authors [Median (IQR)]**	3 (2–5)	3 (2–5)	6 (4–8)

^b^: Median, IQR: interquartile range

### Compliance of reporting to the standard CONSORT and CONSORT extension checklists

Among the 36 items of the standard CONSORT checklist, only four were adequately reported among those trials, including specification of study objectives or hypotheses (84.0%), statement about the eligibility criteria for participants (92.8%), description of study settings and locations (85.2%), and generalizability of the trial findings (87.7%) (Table 2).

**Table 2:** Compliance of reporting to the standard CONSORT and CONSORT extension checklists.

Items	Total (n = 318, %)	Chinese (n = 264, %)	English (n = 54, %)
1.Title	53 (17.7)	19 (7.2)	34 (63.0)
2.Structured abstracts	240 (75.5)	210 (79.5)	30 (55.6)
3.Description of the experimental treatment, comparator, care providers, centers and blinding status^†^	6 (2.0)	0 (0.0)	6 (11.1)
4.Background and rationale	84 (26.4)	42 (15.9)	42 (77.8)
5.Objectives or hypotheses	267 (84.0)	217 (82.2)	50 (92.6)
6.Trial design	29 (9.1)	17 (6.4)	12 (22.2)
7.Changes to methods after trial commencement with reasons	2 (0.6)	1 (0.4)	1 (1.9)
8.Eligibility criteria for participants	295 (92.8)	242 (91.7)	53 (98.1)
9.Eligibility criteria for centers and those performing the interventions^†^	4 (1.3)	0 (0.0)	4 (7.4)
10.Settings and locations	271 (85.2)	234 (88.6)	37 (68.5)
11.Completely defined pre-specified outcomes	26 (8.2)	0 (0.0)	26 (48.1)
12.Changes to trial outcomes after the trial commenced, with reasons	0 (0.0)	0 (0.0)	0 (0.0)
13.How sample size was determined	24 (7.5)	2 (0.8)	22 (40.7)
14.Details of whether and how the clustering by care providers or centers was addressed^†^	0 (0.0)	0 (0.0)	0 (0.0)
15.Interim analyses and stopping guidelines	2 (0.6)	0 (0.0)	2 (3.7)
16.Random allocation sequence	154 (48.4)	129 (48.9)	25 (46.3)
17.How care providers were allocated to each trial group^†^	0 (0.0)	0 (0.0)	0 (0.0)
18.Type of randomization	34 (11.50)	12 (4.5)	22 (68.8)
19.Allocation concealment	31 (10.5)	17 (6.4)	14 (43.8)
20.Implementation of randomization	15 (4.7)	1 (0.4)	14 (25.9)
21.Blinding	45 (14.2)	9 (3.4)	36 (66.7)
22.Co-interventions were blinded to group assignment^†^	0 (0.0)	0 (0.0)	0 (0.0)
23.Similarity of interventions	11 (3.5)	0 (0.0)	11 (20.4)
24.Statistical methods for outcomes	252 (79.2)	206 (78.0)	46 (85.2)
25.Details of whether and how the clustering by care providers or centers was addressed^†^	0 (0.0)	0 (0.0)	0 (0.0)
26.Methods for additional analyses	14 (4.4)	1 (0.4)	13 (24.1)
27.Participant flow diagram	31 (9.7)	0 (0.0)	31 (57.4)
28.Number of care providers or centers and the number of patients treated by each care provider center^†^	0 (0.0)	0 (0.0)	0 (0.0)
29.Losses and exclusions after randomization, together with reason	29 (9.1)	0 (0.0)	29 (53.7)
30.Dates of recruitment and follow-up	246 (77.4)	212 (80.3)	34 (63.0)
31.Why the trial ended or was stopped	5 (1.6)	3 (1.1)	2 (3.7)
32.Baseline data	149 (46.9)	103 (39.0)	46 (85.2)
33.Description of care providers and centers^†^	5 (1.6)	0 (0.0)	5 (9.3)
34.Numbers analyzed	210 (66.0)	173 (65.5)	37 (68.5)
35.For each primary and secondary outcome, results for each group, and the estimated effect size and its precision	78 (24.5)	55 (20.8)	23 (42.6)
36.For binary outcomes, presentation of both absolute and relative effect sizes is recommended			
37.Ancillary analyses	12 (3.8)	0 (0.0)	12 (22.2)
38.Harms	67 (21.1)	34 (12.9)	33 (61.1)
39.Limitations	47 (14.8)	20 (7.6)	27 (50.0)
40.Generalizability	279 (87.7)	227 (86.0)	52 (96.3)
41.Generalizability of the trial findings according to the intervention, comparators and patients, etc^†^	15 (4.7)	0 (0.0)	15 (27.8)
42.Interpretation	146 (45.9)	97 (36.7)	49 (90.7)
43.Take into account the choice of the comparator, lack of or partial blinding, unequal expertise of care providers or centers in each group^†^	14 (4.4)	0 (0.0)	14 (25.9)
44.Registration	14 (4.4)	1 (0.4)	13 (24.1)
45.Protocol	15 (4.7)	1 (0.4)	14 (25.9)
46.Funding	95 (29.9)	65 (24.6)	30 (55.6)
**Summarized scores**			
Standard CONSORT^‡^	12 (10–14)	11 (10–14)	21 (15–25)
CONSORT Extension^‡^	3 (3–5)	3 (3–3)	4 (3–5)

^**†**^: Items related to CONSORT Extension for Trials Assessing Non-Pharmacological Treatments

^**‡**:^ Score was showed as Median (interquartile range)

Poorly reported items (i.e. reporting in less than 5% of trials) were: specification of important changes to methods after trial commencement (0.6%), description of any changes to trial outcomes after the trial commenced with reasons (0.0%), implementation of interim analyses and stopping guidelines (0.6%), statement about why the trial ended or was stopped (1.6%), statement about the registration (4.4%), accessibility of full trial protocol (4.7%), implementation of randomization (4.7%), description of the similarity of interventions (3.5%), conduct of ancillary analyses (3.8%), and presentation of methods for additional analyses (4.4%).

All the ten CONSORT extension items were poorly reported (reported less than 10% of trials), such as description of the experimental treatment, comparator, care providers, centers and blinding status (2%), presentation of eligibility criteria for centers and those performing the interventions or statement about if the co-interventions were blinded to group assignment (0%) (Table 2). Compared with the trials published in Chinese, those published in English journals were more likely to report items related to the implementation of randomization, allocation concealment and blinding, although the adequate reporting of the items were generally poor.

### Compliance of reporting to the STRICTA checklist

The reporting about acupuncture inventions were seriously limited. Items with markedly incomplete reporting (reported in less than 20% of trials) were: description of style of acupuncture (8.5%), statement of reason for the treatment (14.2%), presentation of extent to which treatment was varied (1.3%), description of details of other interventions (18.6%), specification of setting and context of treatment (15.1%), statement of practitioner background (item 61, 7.2%) and rationale for the control (9.1%).

Over 80% of the trials reported items related to the details about needling, including the number of needle insertions per subject per session (82.4%), names of the points used (89.6%), needle retention time (86.5%), number of treatment sessions (90.6%), and frequency and duration of treatment sessions (91.8%). Overall, items related to the details of needling were well reported in both Chinese and English trials (Table 3). The median total scores were 24 (interquartile range 21–27) for the CONSORT plus STRICTA checklists, 12 (interquartile range 10–14) for the standard CONSORT, 3 (interquartile range 3–5) for the CONSORT extension and 9 (interquartile range 8–10) for the STRICTA. Compared with the total score of the 264 trials published in Chinese (median: 23, interquartile range 21–26), those published in English were more likely to have a higher total score (median: 25, interquartile range 28–38).

**Table 3:** Compliance of reporting to the STRICTA checklist.

Items	Total (n = 318, %)	Chinese (n = 264, %)	English (n = 54, %)
**Acupuncture rationale**			
47.Style of acupuncture	27 (8.5)	3 (1.1)	24 (44.4)
48.Reason for the treatment	45 (14.2)	20 (7.6)	25 (46.3)
49.Extent to which treatment was varied	4 (1.3)	2 (0.8)	2 (3.7)
**Details of needling**			
50.Number of needle insertions per subject	262 (82.4)	238 (90.2)	24 (44.4)
51.Names of the points	285 (89.6)	241 (91.3)	44 (81.5)
52.Depth of insertion	151 (47.5)	110 (41.7)	41 (75.9)
53.Responses sought	252 (79.2)	210 (79.5)	42 (77.8)
54.Needle stimulation	241 (75.8)	198 (75.0)	43 (79.6)
55.Needle retention time	275 (86.5)	235 (89.0)	40 (74.1)
56.Needle type	252 (79.2)	209 (79.2)	43 (79.6)
**Treatment regimen**			
57.Number of treatment sessions	288 (90.6)	248 (93.9)	40 (74.1)
58.Frequency and duration of treatment sessions	292 (91.8)	251 (95.1)	41 (75.9)
**Other components of treatment**			
59. Details of other interventions	59 (18.6)	51 (19.3)	8 (14.8)
60.Setting and context of treatment	48 (15.1)	40 (15.2)	8 (14.8)
**Practitioner background**			
61.Practitioner background	23 (7.2)	1 (0.4)	22 (40.7)
**Control or comparator interventions**			
62.Rationale for the control	29 (9.1)	5 (1.9)	24 (65.6)
63.Precise description of the control	265 (83.3)	232 (87.9)	33 (61.1)
**Summarized scores**			
STRICTA^‡^	9 (8–10)	9 (8–10)	9 (7–11)
Total scores ^a^^‡^	24 (21–27)	23 (21–26)	25 (28–38)

^**a**^: The score was calculate based on the items of the standard CONSORT, CONSORT Extension for Trials Assessing Non-Pharmacological Treatments and STRICTA checklist

^**‡**:^ Score was showed as Median (interquartile range)

### Factors associated with the reporting quality

Multivariable linear regression analyses showed that RCTs including authors with expertise in epidemiology or statistics (β coefficient 6.37, 95% confidence interval (CI) 3.49 to 9.24), published in English language (β coefficient 8.35, 95% CI 6.68 to 10.01), and enrolling multiple study sites (β coefficient: 4.80, 95% CI: 2.85 to 6.76) were statistically associated with a higher total score (indicating better overall reporting quality) (Table 4). The beta coefficients of each individual variable corresponded to an increase of 4.80, 6.37 and 8.35 points to the total score, respectively. The analysis with the standard CONSORT score had similar results with the total score (Table 5). Sensitivity regression analyses suggested similar results (S4 File).

**Table 4:** Factors associated with overall reporting quality^a^.

Variables	Univariable analysis	*P*	Multivariable analysis	*P*
	Coefficient (95% CI)		Coefficient (95% CI)	
**The main effect of the primary outcome**				
significant vs non-significant_[ref]_	0.73 (-0.74 to 2.20)	0.33	-0.03 (-1.14 to 1.09)	0.96
**Language**				
English vs Chinese_[ref]_	10.70 (9.14 to 12.27)	<0.001	8.35 (6.68 to 10.01)	<0.001
**Author’s affiliation to statistics or epidemiology department**				
yes versus no_[ref]_	14.55 (11.59 to 17.51)	<0.001	6.37 (3.49 to 9.24)	<0.001
**Center**				
multicenter vs single center_[ref]_	7.74 (5.37 to 10.11)	<0.001	4.80 (2.85 to 6.76)	<0.001
**Sample size**				
>80 vs ≤80_[ref]_	0.47 (-1.01 to 1.94)	0.53	0.20 (-0.92 to 1.33)	0.72

Note: [ref]: reference level

^a^:as indicated by the total score calculated based on the items of the standard CONSORT, CONSORT Extension for Trials Assessing Non-Pharmacological Treatments and STRICTA checklist

**Table 5:** Factors associated with the standard CONSORT.

Variables	Univariable analysis	*P*	Multivariable analysis	*P*
	Coefficient (95% CI)		Coefficient (95% CI)	
**The main effect of the primary outcome**				
significant vs non-significant_[ref]_	0.48 (-0.64 to 1.60)	0.40	-0.15 (-0.97 to 0.67)	0. 72
**Language**				
English vs Chinese_[ref]_	8.55 (7.41 to 9.69)	<0.001	7.00 (5.77 to 8.24)	<0.001
**Author’s affiliation to statistics or** **epidemiology department**				
yes versus no_[ref]_	10.72 (8.46 to 12.97)	<0.001	4.05 (1.92 to 6.18)	<0.001
**Center**				
multicenter vs single center_[ref]_	5.67 (3.87 to 7.47)	<0.001	3.47 (2.02 to 4.92)	<0.001
**Sample size**				
>80 vs ≤80_[ref]_	0.15 (-0.96 to 1.26)	0.79	-0.003 (0.84 to 0.84)	0.99

Note: _[ref]:_ reference level

## Discussion

Our study identified 318 RCTs of acupuncture for KOA. To the best of our knowledge, this is the first study that systematically assessed the extent to which such trials complied with the reporting guidelines in this specific field. Across those RCTs, we found that a considerable number of items were not adequately reported, which may jeopardize the evaluation of internal and external validity of trial results. The apparent low adherence rate is primarily due to the poor reporting; However, the inapplicability of some items (e.g. description of any changes to trial outcomes, implementation of interim analyses and stopping guidelines and statement about why the trial ended or was stopped) to a small proportion of trials may affect the assessment as well.

Our study showed that less than half of the standard CONSORT items were reported. Reporting of items related to methodological domains, such as type of randomization, allocation concealment, and blinding was particularly incomplete. This is likely due to the poor reporting of Chinese RCTs, which accounted for 83% of total reports. Similar to previous report, the methodological quality of most acupuncture trials was generally poor in Chinese journals [15, 43]. The results from our regression analyses also supported that RCTs published in Chinese had lower reporting quality. This finding is consistent with earlier studies addressing reporting quality in other subspecialties [9, 11, 38, 44–46].

Although the STRICTA statement recommends reporting on acupuncture rationale and practitioner background, information related to these items seems to have been largely under-reported. This is in line with the similar studies conducted by Lu and Ma [11, 47]. Since acupuncture is a practitioner-dependent, non-pharmacological complex intervention, adequate reporting of items related to study contexts is essential for readers to determine whether the results of a study apply to their own practice [48]. We would argue that further detail is required for acupuncture interventions, which are often far more complex than drug interventions.

In contrast, our study found that the item related to details of needling materials was well reported (79.2% complete). This is inconsistent with the research published in 2013 that common missing element of non-pharmacological interventions in RCTs were materials [40]. This discrepancy may be because the included studies were limited to trials of acupuncture, and we included CONSORT extension and STRICTA that had items specific to acupuncture intervention [9]. The findings probably reflect increasing awareness and requirement of adopting STRICTA by authors, editorials and peer-reviewers. [48]

The inconsistent and suboptimal reporting across items implied that certain items may have been treated differentially in their importance to authors [9]. Removal of details about interventions (i.e. those related to STRICTA recommendations) from main reports, due to word limitations or suggestions from editors and peer reviewers, might be an external issue limiting the trial reporting [48]. One study showed that the page length was associated with reporting quality [49].

We identified that the author expertise with epidemiology or statistics was associated with higher quality trials. This finding was consistent with an earlier study, in which inclusion of an author who had a cited degree in epidemiology or statistics was almost three times (odds ratio = 2.9) as likely to be of higher quality [50]. Our study also found that trials published in English journals were associated with better reporting [15]. This highlighted that Chinese journals should strictly adopt reporting guidelines, so that transparent, complete and accurate reporting of RCTs can be achieved [49]. Our regression analyses also showed that multicenter studies had better reporting quality. A similar finding was also reported that if RCTs are larger and involve more patients and centers, the reporting quality was improved [51].

### Strengths and limitations

Our study has some strengths. We systematically examined the extent to which randomized trials examining acupuncture intervention for knee osteoarthritis adhered to the CONSORT statement and STRICTA guidelines. We conducted a comprehensive search, developed explicit eligibility criteria, applied rigorous methods for screening studies and collecting data, and used the widely accepted checklists, including the CONSORT statement and STRICTA guideline for assessing quality of reporting.

Our study also has limitations. In assessing quality of reporting, we calculated a quality score, assuming equal weight of each item, although the items of those checklists may carry varying weights (which were yet to be established). We did not use the latest version of the CONSORT extension for non-pharmacological treatments because it was not available when conducting this study. Third, the present study did not investigate the reporting quality of trials published in languages other than Chinese and English; we expected that such studies were very few. Fourth, when scoring the reporting of the items, some items may not be applicable to all trials. For example, those items such as clustering may not be appropriate for a single center comparison; and reporting of interim analyses is not applicable if a trial was not planned such analysis. These situations may have affected our comparison. Nevertheless, both the main and sensitivity analyses showed consistent findings, suggesting that our results are robust across the coding systems.

## Conclusion

The reporting in RCTs of acupuncture for KOA was generally suboptimal. Items related to methodology, acupuncture rationale, practitioner background and comparator interventions remained under-reported in many trials. To improve the reporting quality, journals, especially those published in Chinese, should encourage strict adherence to the CONSORT and STRICTA guidelines.

## Supporting information

S1 File Search strategies. (DOCX)

S2 File The criteria for selecting a primary outcome. (DOCX)

S3 File List of included RCTs. (DOCX)

S4 File The results of sensitivity analyses. (DOCX)

S1 Table The evaluation checklist. (DOCX)
