# Supplementary material for: The quality of reporting in randomized controlled trials of acupuncture for knee osteoarthritis: A cross-sectional survey
Source: PLoS One. 2018 Apr 12;13(4):e0195652. doi: 10.1371/journal.pone.0195652 (PMC5896985; doi:10.1371/journal.pone.0195652)
Supplement: S1 File — (DOCX) [file pone.0195652.s001.docx]

**Search strategies**

**PubMed**

#1 "Osteoarthritis, Knee"[Mesh]

#2 Knee Osteoarthritides[tw]

#3 Knee Osteoarthritis[tw]

#4 "Osteoarthritis"[Mesh]

#5 Osteoarthritides[tw]

#6 Osteoarthrosis[tw]

#7 Osteoarthroses[tw]

#8 Degenerative Arthritides

#9 Degenerative Arthritis

#10 Knee[Mesh]

#11 Knee[tw]

#12 knees[tw]

#13  #1 or #2 or #3

#14 #4 or #5 or #6 or #7 or #8 or #9

#15 #10 or #11 or #12

#16 #14 and #15

#17 #13 or #16

#18 "Acupuncture"[Mesh]

#19 "Acupuncture Therapy"[Mesh]

#20 Acupuncture[tw]

#21 acupoint injection[tw]

#22 trigger point[tw]

#23 dry needling[tw]

#24 electroacupuncture[tw]

#25 electro-acupuncture[tw]

#26 filiform needle[tw]

#27 fire needle[tw]

#28 silver needle[tw]

#29 #18 or #19 or #20 or #21 or #22 or #23 or #24 or #25 or #26 or #27 or #28

#30 Randomized controlled trial[pt] OR Controlled clinical trial[pt] OR Randomized[tiab] OR Placebo[tiab] OR

Clinical Trials as Topic[Mesh] OR Randomly[tiab] OR Trials[ti]

#31 #17 and # 29 and # 30

**Embase**

1 Clinical trial/

2 Randomized controlled trial/

3 Randomization/

4 Single blind procedure/

5 Double blind procedure/

6 Crossover procedure/

7 Placebo/

8 Randomi?ed controlled trial$.tw.

9 Rct.tw.

10 Random allocation.tw.

11 Randomly allocated.tw.)

12 Allocated randomly.tw.

13 (allocated adj2 random).tw.

14 Single blind$.tw.

15 Double blind$.tw.

16 ((treble or triple) adj blind$).tw.

17 Placebo$.tw.

18 Prospective study/

19 or/1-18

20 Case study/

21 Case report.tw.

22 Abstract report/ or letter/

23 or/20-22

24 19 not 23

25 Osteoarthritis, Knee.mp. or exp knee osteoarthritis/

26 knee osteoarthritis.mp. or exp knee osteoarthritis/

27 Knee Osteoarthritis.tw.

28 exp knee osteoarthritis/ or osteoarthritis.mp.

29 Osteoarthrosis.tw.

30 Osteoarthroses.tw.

31 Degenerative Arthritides.tw.

32 Degenerative Arthritis.tw.

33 Knee.mp. or exp knee/ or exp knee arthritis/

34 (knees or Knee).tw.

35 28 or 29 or 30 or 31 or 32

36 33 or 34

37 35 and 36

38 25 or 26 or 27

39 37 or 38

40 24 and 39

41 exp acupuncture analgesia/ or Acupuncture.mp. or exp acupuncture/ or exp acupuncture needle/

42 Acupuncture Therapy.mp. or acupuncture/

43 Acupuncture.tw.

44 exp electroacupuncture/ or exp acupuncture/ or acupoint injection.mp.

45 exp acupuncture/ or trigger point.mp. or exp trigger point/

46 exp trigger point/ or dry needling.mp.

47 electroacupuncture.mp. or exp acupuncture/ or exp electroacupuncture/

48 exp acupuncture/ or exp electroacupuncture/ or electro-acupuncture.mp.

49 exp acupuncture analgesia/ or exp electroacupuncture/ or exp acupuncture/ or filiform needle.mp. or exp needle/

50 exp acupuncture/ or fire needle.mp.

51 exp needle/ or exp acupuncture/ or silver needle.mp.

52 (acupoint injection or trigger point or dry needling or electroacupuncture or electro-acupuncture or filiform needle or fire needle or silver needle).tw.

53 41 or 42 or 43 or 44 or 45 or 46 or 47 or 48 or 49 or 50 or 51

54 52 or 53

55 40 and 54

**Cochrane Central Register of Controlled Trials**

1 Osteoarthritis, Knee.mp. or exp Osteoarthritis, Knee/

2 knee osteoarthritis.mp. or exp Osteoarthritis, Knee/

3 Knee Osteoarthritis.tw.

4 1 or 2 or 3

5 Osteoarthritis.mp. or Osteoarthritis/ or exp Osteoarthritis, Knee/

6 (Osteoarthritides or Osteoarthrosis or Osteoarthroses or Degenerative Arthritides or Degenerative

Arthritis).tw.

7 exp Osteoarthritis, Knee/ or exp Knee/ or Knee.mp.

8 (Knee or knee).tw.

9 5 or 6

10 7 or 8

11 9 and 10

12 11 or 4

13 exp Acupuncture Analgesia/ or exp Acupuncture Therapy/ or exp Acupuncture/ or exp Acupuncture, Ear/

or Acupuncture.mp.

14 Acupuncture Therapy.mp. or exp Acupuncture Therapy/

15 (acupuncture or acupoint injection or trigger point or dry needling or electroacupuncture or electro-

acupuncture or filiform needle or fire needle or silver needle).tw.

16 13 or 14 or 15

17 12 and 16
