# Supplementary material for: The quality of reporting in randomized controlled trials of acupuncture for knee osteoarthritis: A cross-sectional survey
Source: PLoS One. 2018 Apr 12;13(4):e0195652. doi: 10.1371/journal.pone.0195652 (PMC5896985; doi:10.1371/journal.pone.0195652)

**The criteria for selecting a primary outcome**

**Reference**: Bala MM, Akl EA, Sun X, Bassler D, Mertz D, Mejza F, et al. Randomized trials published in higher vs. lower impact journals differ in design, conduct, and analysis. J Clin Epidemiol. 2013;3: 286-295.


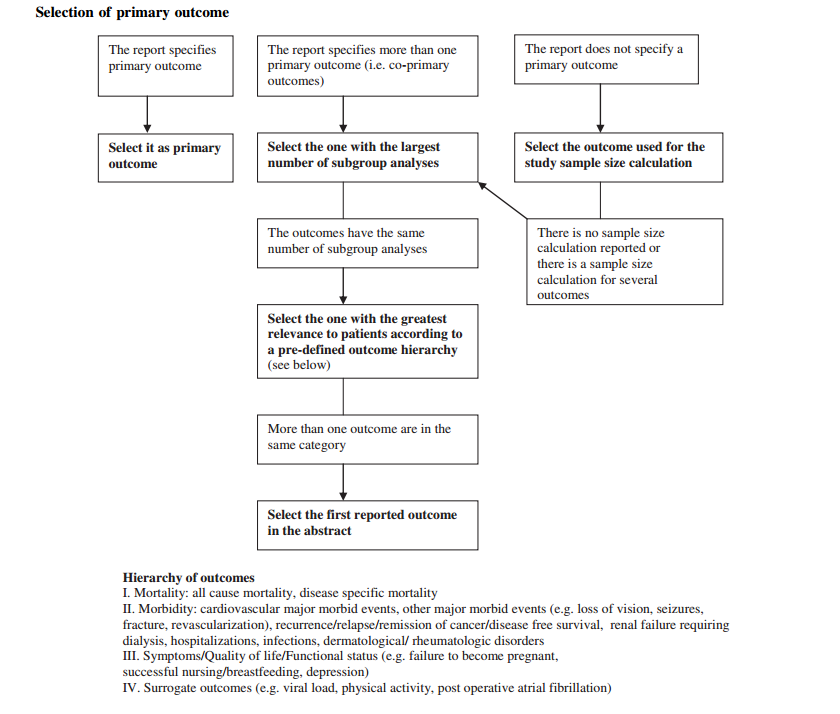

Supplement: S2 File — (DOCX) [file pone.0195652.s002.docx]
