# Supplementary material for: The quality of reporting in randomized controlled trials of acupuncture for knee osteoarthritis: A cross-sectional survey
Source: PLoS One. 2018 Apr 12;13(4):e0195652. doi: 10.1371/journal.pone.0195652 (PMC5896985; doi:10.1371/journal.pone.0195652)
Supplement: S3 File — (DOCX) [file pone.0195652.s003.docx]

**List of included RCTs**

1. J Huang. The effects of moxibustion treatment of knee osteoarthritis: clinical observation on 50 cases. *Journal of Clinical Acupuncture and Moxibustion* 2002;3(18):44-45.

2. L Qiu. The clinical effects of needle acupuncture on knee osteoarthritis *Journal of Sichuan of Traditional Medicine* 2002;11(20):76-77.

3. Z Yao, X Huang, W Zhang. The effects comparison of moxibustion and acupuncture treatment on knee osteoarthritis *Journal of Clinical Acupuncture and Moxibustion* 2003;7(19):32-33.

4. Y Zhang. The clinical observation of acupuncture treatment of knee osteoarthritis. *Modern Journal of Integrated Chinese Traditional and Western Medicine* 2003;22(12):2406-07.

5. L Huang, E Liang. Acupuncture in patients with osteoarthritis of the knee. *Chinese Manipulation & Qi Gong Therapy* 2004;3(20):13-14.

6. X Huang. The clinical effect of needle acupllncture treatment on patients suffer from knee osteoarthritis [Doctor]. Chengdu University of Traditional Chinese Medcine, 2004.

7. B Zhang. Clinical study on treatment of knee osteoarthritis by penetration needling with long needle. *Chinese Acupuncture & Moxibustion* 2004;9(24):613-14.

8. J Jia. Acupuncture combined with functional exercise for the elder patients with knee osteoarthritis. *Chinese Journal of Clinical Rehabilitation* 2005;10(9):18-19.

9. L Lin, Y Liang. Clinical observation on electric heat needle therapy for treatment of gonarthritis. *Chinese Acupuncture & Moxibustion* 2005;10(25):689-90.

10. F Lv. The clinical effect of needle acupllncture and traditional Chinese medcien treatment on patients suffer from knee osteoarthritis. *Heibei Journal of traditional chinese medcine* 2005;1(27):45.

11. B Meng. The effects of electroacupuncture treatment of knee osteoarthritis: clinical observation on 54 cases. *Journal of Medicine & Pharmacy of Chinese Minorities* 2005;1(11):15.

12. CD Li, XY Huang, XG Yang, et al. [Observation on therapeutic effect of warming needle moxibustion on knee osteoarthritis of deficiency-cold type]. *Zhongguo Zhen Jiu* 2006;3(26):189-91.

13. B Chao, A Gu. Clinical Observaton on MYL - I Mogolian Medical Therapy Warm - Acuptmture Instrument Treat Knee- Joint Osteoarthritis. *Journal of Medicine & Pharmacy of Chinese Minorities* 2006;6(12):32-33.

14. Q Kuang. Fire needle treatment of knee osteoarthritis in 50 cases. *Journal of Clinical Acupuncture and Moxibustion* 2006;5(22):19-20.

15. C Li. Observation on therapeutic effect of warming needle moxibustion on knee osteOarthritis of deficiencycold type. *Chinese Acupuncture & Moxibustion* 2006;3(26):189-91.

16. G Lv. Needle therapy treatment on knee osteoarthritis. *Nei Mongol Journal of Traditional Chinese Medicine* 2006;06(39-40.

17. M Qiu, Q Dai, T Chen, et al. Electroacupuncture trratment on knee osteoarthritis. *The Journal of Traditional Chinese Orthopedics and Traumatology* 2006;3(18):15-16.

18. L Zhang. The effect of warm needle and electric acupuncture treatment on knee osteoarthritis [Master]. Shandong University of Traditional Chinese Medicine, 2006.

19. L Cao, W He. Needle knife and triple therapy on knee osteoarthritis: 80 cases of clinical observation. *Jiangsu Journal of Traditional Chinese Medicine* 2007;5(39):39-40.

20. J Cheng, J Mu, L Peng, et al. Laser acuknife for degenerative knee: a clinical observation. *Chinese Journal of Laser Medicine & Surgery* 2007;5(16):313-16.

21. Z Li. Clinical Observation on Treating 60 Cases of Hyperplastic Gonarthritis with Electric -acupuncture and Iontophoresis of TCM. *Guiding journal of traditional Chinese medcine* 2007;4(13):55-56.

22. G Zhang, J Zhou, N Wen. Needling treatment of 34 cases of c knee osteoarthritis. *Chinese Journal of Traditional Medical Science and Technology* 2007;4(14):260.

23. Y Zhang. Study of the clinical efficacy of acupotomy for treating Knee Osteoarthritis：a randomized,controlled trial. *China Science and Technology Information* 2007;18(3):219-20.

24. ZH Wu, F Bao. Observation on therapeutic effects of electroacupuncture for the treatment of knee osteoarthritis. *Zhongguo Gu Shang* 2008;3(21):170-2.

25. H Zeng, B Nie, L Shi. Observation on therapeutic reffect of blood-letting puncture combined with red-hot needle therapy on knee osteoarthritis. *Chinese Acupuncture & Moxibustion* 2008;7(28):493-95.

26. W Chen, M Lin, C Liu. Therapeutic Effect of Acupuncture with Warmed Needle Plus Massage on Ossa Articniaria Osteoarthritis：A clinical report of 60 Cases. *Liaoning Journal of Traditional Chinese Medicine* 2008;11(35):1735-36.

27. Z Zhen. Clinical observation on electric acupuncture therapy for treating gonarthritis. *Acta Academiae Medicinae CPAPF* 2008;4(17):316-17.

28. Z Lu. Warm acupuncture treatment of knee osteoarthritis: 60 cases. *Henan Traditional Chinese Medicine* 2008;12(28):77-78.

29. Y Luo. Acupuncture and Moxibustion on Elderly Patients with Knee Osteoarthritis Estradiol Levels of Clinical Research [Master]. Guangxi University of Chinese Medicine, 2008.

30. W Quan, X Zhang, J Jiang, et al. Acupotomology pressure analysis for the treatment of knee osteoarthritis. *Journal of Chengdu University of Traditional Chinese Medicine* 2008;3(31):6-8.

31. Q Sun. The clinical observation of acupuncture treatment of knee osteoarthritis *Medical Innovation of China* 2008;33(5):30-31.

32. Y Wang, Y Shen. Clinical Effect Observation of Shaoshanhuo Acupuncture in Treatment of Gonarthritis. *chinese Modern Doctor* 2008;26(46):74.

33. H Wu, F Bao. Observation on therapeutic effects of electroacupuncture for the treatment of knee osteoarthritis. *China Journal of Orthopaedics and Traumatology* 2008;3(21):170-72.

34. S Xu. Observations on the Therapeutic Effect of Acupuncture on Genual Osteoarthritis. *Shanghai Journal of Acupuncture and Moxibustion* 2008;4(27):11-12.

35. J Yang. The clinical study on knee steoarthritis treated by warm needle accupuncture [Master]. Nanjing University of Traditional Chinese Medicine 2008.

36. X Zhao, X Lu, Z Li, et al. Warm acupuncture treatment of knee osteoarthritis: 50 cases. *Traditional Chinese Medicine Journal* 2008;4(7):49-50.

37. G Zeng, X Zhang, W Quan, et al. Effects of Needle Knife Relaxing Therapy on Stress Stimulation and Clinical Symptoms of Knee Osteoarthritis. *Chinese Archives of Traditional Chinese Medicine* 2009;1(27):66-68.

38. Y Guo. Clinical Observation on Treatment of Inveterate Knee Osteoarthropathy by the Combination of Both Warming Needle Moxibustion and ChronoMedicine. *Liaoning Journal of Traditional Chinese Medicine* 2009;10(4):1771-72.

39. Y Guo, Y Cao, D Zeng, et al. Warm acupuncture treatment on knee osteoarthritis: 60 cases. *Shaanxi Journal of Traditional Chinese Medicine* 2009;9(2):1210-11.

40. C Guo, S Tong, M Song. Needle knife and cupping treatment on knee osteoarthritis : 60 cases. Wuhan conference of acupuncture. Wunhan, 2009:162-69.

41. K Hong, T Wan, X Hong, et al. Clinical Research of Warming Needle Moxibustion in Treating 30 Cases of Knee Osteoarthritis with Yang Deficiency and Cold Coagulation Syndrome. *Journal of Fujian College of Traditional Chinese Medicine* 2009;2(19):46-49.

42. S Hu. Acupotomology improve the symptoms of knee osteoarthritis. *Jiangxi Medical Journal* 2009;11(44):1093-95.

43. X Ku, S Yang, L Zhu, et al. Comparative Study on Clinical Efficacy Between Electroacupuncture and Tuina in Treating Knee Osteoarthritis. *Shanghai Journal of Traditional Chinese Medicine* 2009;8(43):19-21.

44. H Li. Needle knife technique treatment on knee osteoarthritis: 110 cases. *Journal of Changchun College of Traditional Chinese Medicine* 2009;6(25):891-92.

45. Z Li. Needle knife combined with traditional Chinese medicine treatment of knee osteoarthritis(40 cases of clinical observation). *New Journal of Traditional Chinese Medicine* 2009;9(8):89-90.

46. D Lu, L Yang, S Mei. The effects of fire needle treatment of knee osteoarthritis. Conference of Chinese acupuncture. Hangzhou, 2009:644-46.

47. L Peng. Clinical Observation on Treatment of Senile Genual Osteoarthritis by Warm Needle Moxibustion. *Liaoning Journal of Traditional Chinese Medicine* 2009;10(2):1773-74.

48. W Su. Randomized Controlled Trial of Midnight-midday Ebb-flow Syndrome Differentiation Low-frequency Therapeutic Device for the Treatment of Genual Osteoarthritis. *Shanghai Journal of Acupuncture and Moxibustion* 2009;2(28):95-97.

49. Z Wang. Effect observation of warm acupuncture treatment on knee osteoarthritis. *Chinese Journal of Primary Medicine and Pharmacy* 2009;7(2):1313-14.

50. Y Xuan. Clinical Observations on the Combined Use of Acupuncture and Medicine to Treat Genual Osteoarthritis. *Shanghai Journal of Acupuncture and Moxibustion* 2009;8(28):460-61.

51. Y Zhang. Effect acupuncture treatment on knee osteoarthritis: 32 cases. *Clinical reaseaarch of Chinese Medcine* 2009;21(3):72.

52. Z Zhang. Effect acupuncture treatment on knee osteoarthritis: 40 cases. *GUIDING JOURNAL OF TCM* 2009;4(15):64-66.

53. W Zhou, Z Zhou. Clinical Observation on Treatment of knee osteoarthritis by Warm Needle Moxibustion and cupping. *Rsearch of Integrated Traditional Chinese and Western Medicine* 2009;1(1):37.

54. G Cai. Clinical Study of Micro-invasive Needle Knife Treatment for Genual Osteoarthritis. *Shanghai Journal of Acupuncture and Moxibustion* 2009;2(28):98-99.

55. Q Zhun. Effect observation of acupuncture treatment on knee osteoarthritis. *China's Naturopathy* 2009;8(17):12-13.

56. W Chen, X Chen. Clinical Observation on Treatment of knee osteoarthritis by Warm Needle Moxibustion and Traditional Chinese Medcine. *Massage and rehabilitation medicine* 2010;2(25):8-10.

57. C Guo. Effects of Acupotomy Therapy on Tenderness Point Arounding Knee Joint in Patients with Knee Osteoarthritis：a Randomized Controlled Clinical Trial. *Journal of Chengdu University of Traditional Chinese Medicine* 2010;3(3-5.

58. Y Jia. Clinical Observation on Treatment of knee osteoarthritis by electroacupuncture and Traditional Chinese Medcine. *Journal of Traditional Chinese Medicine* 2010;7(1236-37.

59. B Liu, X Liu, H Wang. Clinical Observations on Electroacupuncture plus Injections of Sodium Hyaluronate for the Treatment of Knee Osteoarthritis. *Shanghai Journal of Acupuncture and Moxibustion* 2010;9(29):593-95.

60. W Quan, X Zhang. Effect of acuscalpel on relaxing soft tissue tension around the osteoarthritis knee. *Medical Journal of the Chinese People＇s Armed Police Forces* 2010;11(21):945-47.

61. J Wang, Y Wang, Y Lu, et al. Analysis of the Therapeutic Effect of Warm Point-to-point Needling on Senile Knee Osteoarthritis. *Shanghai Journal of Acupuncture and Moxibustion* 2010;6(29):390-92.

62. Y Wei. Clinical Observation of Hooking Therapy for Serous Osteoarthritis of Knee Joint. *World Journal Of Integrated Traditional and Wesrern Medicine* 2010;10(5):889-91.

63. J Wu, W Zhang. Clinical Observation on Electroacupuncture for Treatment of Knee Osteoarthritis. *Journal of Clinical Acupuncture and Moxibustion* 2010;8:38-39.

64. N Yan, M Lu, B Zhang. The effects of arthroscopy cleaning with sodium hyaluronate treatment on knee osteoarthritis. *Guide of China Medicine* 2010;29(8):111-12.

65. W Yan, J Yin, D Li, et al. Acupoint sticking therapy knee osteoarthritis: 50 cases *Jiangsu Journal of Traditional Chinese Medicine* 2010;6(42):50-51.

66. W Yan, G Li, L Li, et al. Variable-frequency electroacupuncture for elderly patients with knee osteoarthritis. *Chinese Journal of Physical Medicine and Rehabilitation* 2010;4(4):285-88.

67. X Yan, Y Zhang, L Yu, et al. Comparison of the efficacy of electroacupuncture and warming-promotion of acupuncture in patients with osteoarthritis of knee. *China Journal of Rehabilitation Medicine* 2010;5:447-50.

68. G Zhao. Short-term observation of Hydro--acupuncture for female knee osteoarthritis [Master]. Fujian University of Traditional Chinese Medicine, 2010.

69. W Zhao. The effects of fire acupuncture and Chinese Medcine treatment on knee osteoarthritis. *Chinese Journal of Ethnomedicine and Ethnopharmacy* 2010;9(19):167.

70. H Zhu. Treatment of 56 Cases of Knee Osteoarthritis with Therapy of Fire-needle and Cupping. *Journal of Nanjing University of Traditional Chinese Medicine（Natural Science）* 2010;6(26):475-76.

71. M Fu, Z Zhang. Knee osteoarthritis treated with acupuncture at the points selected according to syndrome differentiation: a randomized controlled trial. *Zhongguo Zhen Jiu* 2011;12(31):1062-6.

72. B Chen, Z Fang, F Xiong. A Comparative Study on The Impact of Clinical Symptoms of Patients with Knee Osteoarthritis Cured by Moxibustion or Acupuncture and Their Treatment Charataristics Studied Through Inquiry. *Liaoning Journal of Traditional Chinese Medicine* 2011;6(38):1186-88.

73. M Chen, X Chen, Y Gu, et al. Treatment of 56 Cases of Knee Osteoarthritis with Therapy of Fire-needle and Cupping. *Journal of Nanjing University of Traditional Chinese Medicine（Natural Science）*2011;4(27):384-86.

74. L Cui. Knee osteoarthritis treated with acupuncture: a randomized controlled trial. *Journal of Sichuan of Traditional Medicine* 2011;7(29):109-10.

75. B DONG, Y Han, C Li, et al. Clinical study on treatment of knee osteoarthritis with long-round needle acupuncture release：a blind,multicenter randomized control study. *China Journal of Traditional Chinese Medicine and Pharmacy* 2011;8(26):1883-85.

76. Y Fan. Cost effect analysis of cupping treatment on knee osteoarthritis. *Chinese Medicine Modern Distance Education of China* 2011;4(9):46-48.

77. C Guo. Clinical study on treatment of knee osteoarthritis with acupotomy. International acupotomy conference Beijing, 2011:14-17.

78. Q Hu, Q Hu, C Jia, et al. Randomized Controlled Clinical Trials of Red-hot Filiform Needle Puncturing for Knee Ostarthritis and Inflammatory Cytokines in Knee Articular Fluid in Senile Knee Ostarthritis Patients. *Acupuncture Research* 2011;2(36):110-15.

79. L Ji, B O. Clinical Observation on Electroacupuncture for Knee Osteoarthritis. *Shanghai Journal of Acupuncture and Moxibustion* 2011;9(30):620-21.

80. B Li. Clinical study on treatment of knee osteoarthritis with fire needle. *Beijing Journal of Traditional Chinese Medicine* 2011;12(30):923-25.

81. C Li, Y Han, B Dong. Clinical Observation on Electroacupuncture for Treatment of Knee Osteoarthritis. The conference of acupuncture and moxibustion. Beijing, 2011:339-45.

82. Y Li, M Zhao. The effects of acupuncture treatment on knee osteoarthritis. The conference of acupuncture and moxibustion. Guangdong, 2011:202-05.

83. Z Li, S Zhen. Clinical Observation of Acupotomy in the Treatment of Knee Osteoarthritis. *Journal of Henan University of Chinese Medicine* 2011;2(26):141-43.

84. J Lin. The clinical efficacy of treating knee osteoarthritis with fire needle and acupuncture respectivel [Master]. Nanjing University of Traditional Chinese Medicine, 2011.

85. M Liu. Curative effect of warm acupuncture combined with ultrashort wave in 100 patients with knee osteoarthritis. *Tianjin Journal of Traditional Chin Medicine* 2011;6(28):469-71.

86. D Lu, Q Wang, S Mei. Clinical Effect of Fire Needle and Warm Needle in Treating Knee Osteoarthritis. *New Journal of Traditional Chinese Medicine* 2011;10(43):94-96.

87. X Ma, J Li, Z Li. Fire acupuncture treatment of knee osteoarthritis: 39 cases. *Journal of Emergency in Traditional Chinese Medicine* 2011;2(20):301-02.

88. R Peng, J Weng, C Liang. Clinical Observation of Acupotomy in the Treatment of Knee Osteoarthritis. *Clinical journal of traditional chinese medicine* 2011;5(23):

89. L Qiu, J Zhai, D Liu, et al. The Curative Effect Observation on Pain Symptom of Patients with Knee Osteoarthritis Living Board Room After Earthquake by Warming Needling Combined with Muscle-strengthening Exercise. *Liaoning Journal of Traditional Chinese Medicine* 2011;4(38):722-24.

90. F Zhu, Y Zhao, X Yin. Acupuncture and complex thorn diarrhea treatment on knee osteoarthritis. *Zhejiang Journal of Traditional Chinese Medicine* 2011;11(46):832-33.

91. H Wang, S Cheng, W Li, et al. Randomly Controlled Trial of Silver Needles plus Sodium Hyaluronate for the Treatment of Knee Osteoarthritis. *Shanghai Journal of Acupuncture and Moxibustion* 2011;4(30):250-51.

92. X Wamg, X Zhang, Y Shen, et al. Randomly Controlled Trial of Silver Needles plus Sodium Hyaluronate for the Treatment of Knee Osteoarthritis. *Shanghai Journal of Acupuncture and Moxibustion* 2011;8(22):707-08.

93. J Wen, Z Yu, C Liang, et al. Massage and acupuncture treatment for early knee osteoarthritis: randomized controlled study. The 18th annual national combine traditional Chinese and western medicine orthopedics academic symposium proceedings. Tianjin, 2011:153-54.

94. Z Xu, T Liu. Knee osteoarthritis treated with electroacupuncture and Traditional Chinese : a randomized controlled trial. *Chinese Medicine Modern Distance Education of China* 2011;2(9):64-65.

95. Z Yu. Massage and acupuncture treatment for knee osteoarthritis [Doctor]. China Academy Chinese medical Science, 2011.

96. X Zhang, W Quan, S Peng, et al. Effects of acupotomy on the footplate pressure and X ray manifestations in the treatment of knee osteoarthritis. *Medical Journal of the Chinese People Armed Police Forces* 2011;10(22):872-75.

97. Y Zhang, Z Zhang, T Gao. Warm acupuncture and massage treatment of 50 cases of knee osteoarthritis. *Shandong Journal of Traditional Chinese Medicine* 2011;7(30):488-89.

98. Y Zhang, D Tong, Z Hou, et al. Therapeutic Effect Evaluation of Warming Needling Therapy for Kidney-deficiency Knee Osteoarthritis by Far-infrared Thermal Texture Maps System. *New Journal of Traditional Chinese Medicine* 2011;4(43):92-94.

99. Y Zhang, Z Wang. The effects of electric acupuncture combined with intra-articular injection of sodium hyaluronate treatment on knee osteoarthritis. *Journal of Chinese Physician* 2011;9(39):49-51.

100. Z Zhang, H Yao, J Chen, et al. Electric acupuncture treatment of early knee osteoarthritis. *Journal of Traditional Chinese Medicine* 2011;8(26):1616-18.

101. W Zhen, Q Yu. Ultrashort wave combined with electroacupuncture treatment for knee osteoarthritis. *Massage and rehabilitation medicine* 2011;23(2):192.

102. N Deng, QF Ran, T Jin, et al. Effect of stiletto needle intervention combined with massage on dysfunction and pain of knee-joints in patients with knee osteoarthrosis. *Zhen Ci Yan Jiu* 2012;1(37):59-63, 76.

103. J Gao, BS Ouyang, Y Zhang, et al. Comparison of the clinical therapeutic effects between electroacupuncture and warming needle moxibustion for knee osteoarthritis of kidney deficiency and marrow insufficiency pattern/syndrome. *Zhongguo Zhen Jiu* 2012;5(32):395-8.

104. L Ying, Y Li, Z Shaofeng, et al. Clinical efficacy research of acupuncture treatment of knee osteoarthritis. Proceedings of the third international symposium on abdominal needle. Beijing, 2012:124-28.

105. T Che, MLQiu, J Sun, et al. Observation on the Treatment Effect of Electroacupuncture for Knee Osteoarthritis. *Shanghai Journal of Acupuncture and Moxibustion* 2012;8(31):595-96.

106. R Du. Clinical Observation of the Transient Analgesic Effect with Bloodletting Therapy on the Treatment of Knee Osteoarthrit [Master]. Shandong University of Traditional Chinese Medicine, 2012.

107. G Chen, R Gu, D Xu. The application of electroacupuncture to postoperative rehabilitation of total knee replacement. *Chinese Acupuncture ＆ Moxibustion* 2012;4(32):309-12.

108. W Fang. Efficacy of electroacupuncture combined with foment bag compress in the treatment of knee osteoarthritis. *Pain Clinic Journal* 2012;6(8):430-32.

109. X Fu. Fu's Subcutaneous Needling’s effect in treating osteoarthritis in the early and middle stage [Master]. Shandong University of Traditional Chinese Medicine 2012.

110. J Gao, B OY, Y Zhang. Comparison of the clinical therapeutic effects between electroacupuncture and warming needle moxibustion for knee osteoarthritis of kidney deficiency and marrow insufficiency pattern/syndrome. *Chinese Acupuncture ＆ Moxibustion* 2012;5(32):395-98.

111. C Guo, T Si, J WEN, et al. Effects of acupotomy therapy on the pain symptoms in patients with knee osteoarthritis：a randomized controlled clinical trial. *Tianjin Journal of Traditional Chin Medicine* 2012;1(29):35-38.

112. C GUO, J Wen, X Zhang, et al. Effect of Acupotomy on Soft Tissue Tension in Patients with Knee Osteoarthritis. *Chinese Journal of Information on Traditional Chinese Medicine* 2012;4(19):15-17.

113. C G, T SI, Y Zhang. Needle knife treatment of knee osteoarthritis Proceedings of the tenth prevention and treatment of diseases of TCM academic communication. Shanmen, 2012:115-17.

114. W Huang. Clinical study of near-term efficacy of acupuncture on treating early knee arthritis. *Clinical Journal of Chinese Medicine* 2012;21(4):47-48.

115. X Li, X Chen, N Jin, et al. Comparison of the Efficacy of Acupuncture with HeNe Laser Light in Treating Knee Osteoarthritis. *Shanghai Journal of Acupuncture and Moxibustion* 2012;11(31):829-30.

116. C Liang, Z Yu, L Yan, et al. Random-controlled Study on Six-Step Manipulation and Electroacupuncture for Early Knee OsteoarthriUs. *Journal of Traditional Chinese Medicine* 2012;17(53):1478-81.

117. L Liu, Y Liu, F CI. Clinical observation of electroacupuncture and massage treatment on knee osteoarthritis *Chinese Medical Journal of Metallurgical Industry* 2012;4(29):474-76.

118. X Liu. Clinical observation of warm needle treatment of knee osteoarthritis. *Modern Traditional Chinese Medicine* 2012;4(32):52-53.

119. L Mao, L Wang, B Xiang. Treating 84 cases of osteoarthritis of the knee by fire needle therapy. *Clinical Journal of Chinese Medicine* 2012;23(4):58-59.

120. L Shen. Clinical Effect Observation of Acupuncture and Moxibustion in the Treatment of Osteoarthritis of the Knee. *Friend of Chemical Industry* 2012;35(31):16,18.

121. Z Shi. Observations on the Efficacy of Contralateral Meridian Needling and Short Thrust Needling plus Functional Training in Treating Knee Osteoarthritis. *Shanghai Journal of Acupuncture and Moxibustion* 2012;11(31):826-28.

122. M Su. The clinical effect for Short Thrust Needling treatment of knee osteoarthritis. *Guide of China Medicine* 2012;34(10):611-12.

123. C Su. Electroacupuncture and injection hyaluronic of acid treatment on the patients with knee osteoarthritis. *The Journal of Practical Medicine* 2012;15(28):2546-48.

124. C Teng. Comparative efficacy of warm acupuncture and electro-acupuncture on treating KOA. *Clinical Journal of Chinese Medicine* 2012;12(4):34-35.

125. J Wang. Massage treatment on knee osteoarthritis. *Guide of China Medicine* JG Wang;3(10):231-32.

126. C Wu. The Clinical Research of warming needle moxibustion on knee osteoarthritis [Master]. Henan University of Chinese Medicine, 2012.

127. D Xia, W Huang, X Wang, et al. Clinical observation of needle warming moxibustion combined with cluster acusector on patients with knee osteoarthritis. *Journal of Traditional Chinese Medicine University of Hunan* 2012;11(32):74-77.

128. L Zhang. Clinical Research of Injection Acumen with Moxibustion in the Treatment of Regressive Gonarthritis. *Journal of Henan University of Chinese Medicine* 2012;5(27):645-46.

129. M Zhang, L Xu, L Xu, et al. PingHengZhen and warmed needle treatment on knee osteoarthritis : 40 cases *Nei Mongol Journal of Traditional Chinese Medicine* 2012;21(31):66-67.

130. M Zhang, L Xu, L Zhao, et al. Observation of the curative effect on balance acupuncture combined with needle warming therapy in the treatment of knee osteoarthritis. *China Medical Herald* 2012;22(9):119-20,26.

131. H Zhong. Clinical observation of warm needle with cupping treatment on knee osteoarthritis: 42 cases. *GUIDING JOURNAL OF TCM* 2012;7(18):70,73.

132. F Bao, Y Zhang, ZH Wu, et al. [Efficacy observation on knee osteoarthritis treated with electroacupuncture and its influence on articular cartilage with T2 mapping]. *Zhongguo Zhen Jiu* 2013;3(33):193-7.

133. GW Cai, J Li, XJ Xu, et al. [Effect of acupoint heat-sensitive moxibustion intervention on serum osteopontin and matrix metalloproteinase-3 in patients with acute knee pain]. *Zhen Ci Yan Jiu* 2013;6(38):488-92.

134. L Qiu, JW Kan, X Zheng, et al. [Observation on the long-term efficacy of knee osteoarthritis treated with warm needling and rehabilitation training]. *Zhongguo Zhen Jiu* 2013;3(33):199-202.

135. H Chao, Y Zhu. Acupuncture combined with Zusanli（ST36） moxibustion therapy on knee osteoarthritis. *Journal of Ningxia Medical College* 2013;10(3):1184-86.

136. Y Chang, H Chang, Y Liu, et al. The effects of needle knife therapy on the C-reactive protein in patients with knee osteoarthritis. *Hebei Journal of Traditional Chinese Medicine* 2013;12(35):1846-47.

137. C Cheng, J Wen, X Lin, et al. Clinical Study on Electro-acupuncture for the Functional Rehabilitation of the Knee Osteoarthritis after Treated with Arthroscope. *Chinese Journal of Traditional Medical Traumatology ＆ Orthopeics* 2013;2(21):26-28.

138. J Cheng. Clinical Observation of Floating Needle Therapy Combined with Sodium Hyaluronate Injection on Treating 52 Cases of Knee Osteoarthritis. *Journal of Sichuan of Traditional Medicine* 2013;7(31):122-24.

139. W Cong, H Dong, J Fang. herapeutic Observation on Electroacupuncture plus Warm Needling Therapy for Knee Osteoarthritis. *Shanghai Journal of Acupuncture and Moxibustion* 2013;3(32):200-02.

140. H Cui. Effect analysis of warm acupuncture treatment for knee osteoarthritis. *Guide of China Medicine* 2013;14(11):669-70.

141. C Fan. Effect of Fire Needling on the Living Quality of Patients with Knee Osteoarthritis. *Shanghai Journal of Acupuncture and Moxibustion* 2013;1(32):41-43.

142. N Fu. Treating knee osteoarthritis with dialectical acupoint selection and electro-acupuncture： a randomized controlled study. *Traditional Chinese Medcine of Tianjing* 2013;10(30):597-600.

143. Y Gao, J Yao, J Guo. Clinical observation on fire needles at bones combined with copping and Tuina for knee osteoarthritis. *Chinese Acupuncture ＆ Moxibustion* 2013;8(33):697-99.

144. Y Guo. Warm acupuncture curative effect analysis for the treatment of knee osteoarthritis. *Guide of China Medicine* 2013;32(11):504-05.

145. K He. Clinical observation of silver needle treatment of knee osteoarthritis: 90 cases. *Yunnan Journal of Traditional Chinese Medicine and Materia Medica* 2013;1(34):45-46.

146. K Huang, M Niu, B Yang, et al. Warming Needle Moxibustion in the Treatment of Knee Osteoarthritis. *Journal of Clinical Acupuncture and Moxibustion* 2013;1(2):48-50.

147. K Huang. Fire needle combined with cutem therapy on knee osteoarthritis: 41 cases *China's elderly health care medicine* 2013;3(11):11-12.

148. P Huang, J Zeng. Clinical Study of Thermal Moxibustion Combined with Traditional Chinese Medicine Ion-introduction for Knee Osteoarthritis. *Journal of Clinical Acupuncture and Moxibustion* 2013;7(2):17-19.

149. Z Ji, P Liu, L Ding, et al. The tendons acupuncture treatment on knee osteoarthritis *Journal of Practical Traditional Chinese Medicine* 2013;9(1):751-52.

150. R Li, Y Han, B Dong. Research of RANTES and MCP-1 Expressions in Knee Osteoarthritis after Acupuncture Moxibustion and Massage Treatments. *Journal of Liaoning University of Traditional Chinese Medicine* 2013;10(15):79-81.

151. X Li, X Tian, W Li. The clinical effect of acupotomy treatment on knee osteoarthritis. Acupotomology branch of China association of Chinese medicine 2013 academic essays. Lanzhou, 2013:194-98.

152. R Liang, B Dong, Z You, et al. Clinical Observation of KOA Treated with Warm Needling Through Meridians A-shi Acupoints. *Journal of Liaoning University of Traditional Chinese Medicine* 2013;10(15):101-03.

153. M Liu, J Mu, S Zhen, et al. Curative Effect Observation on Triple Therapy Treatment of Knee Osteoarthritis. *Journal of Liaoning University of Traditional Chinese Medicine* 2013;8(15):217-19.

154. M Liu, J Mu, K Wang, et al. Therapeutic Observation on Heat-sensitive Moxibustion plus Functional Training for Knee Osteoarthritis. *Shanghai Journal of Acupuncture and Moxibustion* 2013;9(32):750-52.

155. M Liu. Floating needle and functional training treatment on knee osteoarthritis. *Journal of Changchun College of Traditional Chinese Medicine* 2013;3(2):487-88.

156. G Ma, T Wei, T Chen. Fire acupuncture treatment of knee osteoarthritis: 86 cases. *Jilin Journal of Traditional Chinese Medicine* 2013;10(3):1052-53.

157. H Qi, S Chen, L Min. The Effect of He-Ne Laser on Knee Osteoarthritis by Acupoints Irradiation, A Randomized Controlled Trial. *Applied Laser* 2013;6(33):

158. D Qiu, Y Zhang. Warm acupuncture treatment on knee osteoarthritis: 34 cases. *Shaanxi Journal of Traditional Chinese Medicine* 2013;10(2):1398-99.

159. L Qiu, J Yan, X Zhen, et al. Observation on the long-term efficacy of knee osteoarthritis treated with warm needling and rehabi- litation training. *Chinese Acupuncture ＆ Moxibustion* 2013;3(33):199-202.

160. G SQ. Silver needle heating cabinet with sodium hyaluronate injection treatment on knee osteoarthritis: 35 cases. *Global Chinese Medicine* 2013;4(6):286-88.

161. S Tao, T He, Y Luo, et al. Observations on the Efficacy of Fire Acupuncture plus Filiform Needle Acupuncture in Treating Knee Osteoarthritis. *Shanghai Journal of Acupuncture and Moxibustion* 2013;9(32):742-46.

162. C Tong, H Song. Floating needle with traditional Chinese medicine iontophoresis treatment on knee osteoarthritis. *Modern Journal of Integrated Chinese Traditional and Western Medicine* 2013;19(22):2086-87.

163. K Wang, M Liu, S Zhen. Curative Observation of Combined Therapy of Knee Osteoarthritis. *Journal of Sichuan of Traditional Medicine* 2013;7(31):100-02.

164. Y Wang, C Li, Y Lin, et al. Ultrashort wave and electric acupuncture point injection for the treatment of knee osteoarthritis *Zhejiang Journal of Traditional Chinese Medicine* 2013;11(2):834-35.

165. X Yan, W Zhang, B Dong. Warm Acupuncture Treatment of Knee Osteoarthritis Randomized Controlled Study. *Practical internal medicine of traditional Chinese medicine* 2013;4(3):128-30.

166. C Zhang. Needle combined TCM internal treatment on knee osteoarthritis: 60 cases. *Henan JOurnal of Surgery* 2013;6(19):100-01.

167. D Zhang. Observation on Curative Effect of Fu's Subcutaneous Needling with Ultrashort Wave Treated Knee Osteoarthritis. *Chengdu University of Traditional Chinese Medcine* 2013;4(2):52-55.

168. G Zhang. Fire and warm acupuncture treatment on knee osteoarthritis. *Shanghai Journal of Acupuncture and Moxibustion* 2013;4(32):52-55.

169. H Zhang, L Ping, Q Wu. Warm acupuncture combined with cupping treatment on knee osteoarthritis *Hunan Journal of Traditional Chinese Medicine* 2013;5(2):85-87.

170. L Zhang, K Chen, H Wang, et al. The needle knife release technique with sodium hyaluronate injection treatment of knee osteoarthritis. *Chinese JOurnal of Trauma and Disability Medicine* 2013;8(21):136-38.

171. X Zhang, T Huang, A Sun, et al. Observations on the Efficacy of Yang＇s Needle Tapping-cupping Bloodletting Method in Treating Knee Osteoarthritis. *Shanghai Journal of Acupuncture and Moxibustion* 2013;9(32):753-54.

172. Y Zhang. Clinic Effect Comparison of Electroacupuncture and Moxibustion for Knee Osteoarthritis of Kidney and Marrow Deficiency. *Medical Recapitulate* 2013;10(19):1903-05.

173. H Zhao, J Kong, W Lu, et al. Clinical Effects of Acupuncture and Moxibustion in Treating Knee Osteoarthritis. *Acta Universitatis Traditionis Medicalis Sinensis Pharmacologiaeque Shanghai* 2013;2(3):45-47.

174. J Zhou. Effect of fast acupuncture therapy on knee osteoarthritis. *The Journal of Traditional Chinese Orthopedics and Traumatology* 2013;12(25):28-30.

175. L Zhu. Clinical observation of fire needle and cutem therapy on knee osteoarthritis *Beijing Journal of Traditional Chinese Medicine* 2013;8(32):605-07.

176. J Zhu, GW ANG, X Jin, et al. Clinical observation of electro-acupuncture efficacy of early and mid-term senile knee osteoarthritis. *China Journal of Traditional Chinese Medicine and Pharmacy* 2013;7(28):2085-87.

177. G Cheng, W Cheng, YG Jiang. Controlled observation of clinical efficacy on knee osteoarthritis treated with auricular electroacupuncture and the isolated moxibustion with Lingxian herbal paste. *Zhongguo Zhen Jiu* 2014;3(34):230-4.

178. H Chang, Y Hong, Y Liu, et al. Effect of small needle knife therapy on TNF-αand NO in synovial fluid of patient with knee osteoarthritis. *Hebei Journal of Traditional Chinese Medicine* 2014;1(36):78-79,105.

179. Y Chang, H Chang, Y Liu, et al. Small needle knife therapy knee osteoarthritis: 60 cases. *Shandong Journal of Traditional Chinese Medicine* 2014;7(4):557-58.

180. Z Chen, C Dong, C Liang, et al. Clinical study on the acupuncture therapy for treating knee osteoarthritis by inserting needles in Dong’s ex-traordinary points. *The Journal of Traditional Chinese Orthopedics and Traumatology* 2014;11(26):12-14.

181. Y Gao, Q Wang, B Liu, et al. Observation of needle knife combined with local anesthesia on the treatment of knee osteoarthritis. *Hebei Journal of Traditional Chinese Medicine* 2014;12(36):1831-33.

182. W Gu. Curative effect observation of traditional Chinese medicine fumigation and acupuncture for the treatment of knee osteoarthritis. *Chinese journal of practical rural doctors* 2014;24(0):41-42.

183. T He, Y Song, J Ding, et al. Effect of Fire Needling plus Filiform Needles on the Quality of Life in Knee Osteoarthritis Patients. *Shanghai Journal of Acupuncture and Moxibustion* 2014;12(33):1156-59.

184. N Hu, C Liu, H Chen. Clinical observation of warm acupuncture treatment on knee osteoarthritis. *Medpharm ＆ Health* 2014;5(27):174-75.

185. J Huang, L Yang. Clinical Observation of Electro-acupuncture Combined with Oral Glucosamine Hydrochloride Capsules for the Treatment of Knee Osteoarthritis. *West China Medical Journal* 2014;1(29):30-33.

186. J Huang, Z Fu. Warm acupuncture combined with stab winding cutem therapy on knee osteoarthritis: 48 cases. *Nei Mongol Journal of Traditional Chinese Medicine* 2014;24(33):54-55.

187. S Jiang, H Yang, H Zhu, et al. Affect of electrical acupuncture plus massage on quality of life of knee osteoarthritis patients. *WORLD CLINICAL DRUGS* 2014;1(35):25-28.

188. L Li, M Wu, S Chen, et al. Clinical Effect of Acupuncture Treatment on Knee Osteoarthritis Patients with Different Syndromes. *Yunnan Journal of Traditional Chinese Medicine and Materia Medica* 2014;4(35):44-47.

189. W Li. Randomized Controlled Observation of Acupuncture Treatment for Knee Osteoarthritis. *Shanghai Journal of Acupuncture and Moxibustion* 2014;10(33):937-40.

190. X Li, E Liu, W Gao. Electroacupuncture with quadriceps muscle strength training treatment knee osteoarthritis: 60 cases *Chinese JOurnal of Trauma and Disability Medicine* 2014;8(22):47-48.

191. Z Liang, Y Hu, Y Ding, et al. Acupotomology with Chinese herb for the treatment of knee osteoarthritis. *Medical Aesthetics and Cosmetology* 2014;6(0):121-22.

192. Z Liang, Y Hu, Y Ding, et al. Acupotomology treatment of knee osteoarthritis. *Medical Aesthetics and Cosmetology* 2014;5(0 ):132-32.

193. C Liu, X Zhang, H Ji, et al. Acupuncture and moxibustion treatment on knee osteoarthritis. *Journal of Traditional Chinese Medicine* 2014;4(3):770-71.

194. J Liu. Warm acupuncture treatment of knee osteoarthritis *Nei Mongol Journal of Traditional Chinese Medicine* 2014;23(33):52.

195. J Lu, S Ou. Comparison of Clinical Effects between Acusector and Warm Needling Moxibustion in Treating Knee Osteoarthritis of Blood Stasis Obstruction Pattern. *Gansu Journal of Traditional Chinese Medicine* 2014;4(27):119-21.

196. J Lu. Electricacupuncture and warm acupuncture on kidneymarrow deficient type of knee osteoarthritis clinical curative effect and the influence of serum levels of cytokines [Master]. Nanjing University of Traditional Chinese Medicine, 2014.

197. X Ma. Warm acupuncture plus kerotherapy treatment on knee osteoarthritis：40 cases *Hunan Journal of Traditional Chinese Medicine* 2014;2(2):75-76.

198. S Niu, F Zeng. Warm needle treatment on knee osteoarthritis. *Hainan Medical Journal* 2014;18(25):2763-64.

199. C Ren, C Xia. Moxibustion therapy treatment on knee osteoarthritis. *Modern Journal of Integrated Chinese Traditional and Western Medicine* 2014;7(23):754-55.

200. C Ruan, X Chen. erapeutic Effect of Electroacupuncture on Knee Osteoarthritis and Its Impact on Femoral Quadriceps Surface Electromyogram Signals. *Shanghai Journal of Acupuncture and Moxibustion* 2014;8(33):745-47.

201. H Ruan, Z Huang. Effect observation of electroacupuncture treatment of knee osteoarthritis. *Hubei Journal of Traditional Chinese Medicine* 2014;1(36):56-57.

202. J Shang. Acupuncture combined sodium hyaluronate injection drug treatment on knee osteoarthritis. *Chinese Journal of Pain Medicine* 2014;11(20):832-34.

203. H Shao. Clinical control study of gonarthritis caused by yang deficiency and cold coagulation under the therapy of Hes medicine moxibustion [Master]. Gansu college of traditional chinese medicine, 2014.

204. Y Sun. The application of the triple therapy in patients with knee osteoarthritis. *China Medical Engineering* 2014;02(1):30.

205. Z Tang. The clinical curative effect of fire needle treatment on knee osteoarthritis. *Guide of China Medicine* 2014;34(12):294-95.

206. C Wang, Y Wu, S Kou, et al. Clinical Study of Joint Needling plus Warm-unblocking Needling Method for the Treatment of Knee Osteoarthritis. *Shanghai Journal of Acupuncture and Moxibustion* 2014;10(33):935-36.

207. B Wang. Clinical observation on treating degenerative arthritis of knee joint by warming acupuncture and moxibustion plus exercise therapy. *Chinese Journal of Clinical Research* 2014;28(6):35-37.

208. L Wang. Clinical observation on treating Yinxu type of knee osteoarthritis （KOA） by warming acupuncture. *Chinese Journal of Clinical Research* 2014;23(9):29-30.

209. W Wang, W Zhang, J Wang, et al. Therapeutic Analysis of Acupuncture plus Intelligent Chinese Herbal Fumigation for Knee Osteoarthritis. *Shanghai Journal of Acupuncture and Moxibustion* 2014;2(33):165-67.

210. X Wei, D Zhang. Overall Acupotomy-Relaxing Operation with Kinesitherapy Clinical Curative Effect for the Treatment of Patients with Knee Osteoarthritis. *Asia-Pacific Traditional Medicine* 2014;9(10):80-83.

211. B Wu. Electric acupuncture treatment on knee osteoarthritis: 33 cases. *Shandong Journal of Traditional Chinese Medicine* 2014;9(9):750-52.

212. J Wu, C Tian. The clinical curative effect of fire needle treatment on knee osteoarthritis. *Chinese Medicine Modern Distance Education of China* 2014;10(7):61-62.

213. Y Wu. Warm acupuncture and articular cavity injection of sodium hyaluronate treatment on knee osteoarthritis *Yiayao Qianyan* 2014;28(3):177-78.

214. C Yang. Warm acupuncture combined electric acupuncture auxiliary treating of knee osteoarthritis *Chinese Journal of Basic Medicine In Traditional Chinese Medicine* 2014;11(20):1550-51.

215. D Yang, W Tian. Applied in the community warm acupuncture treatment of 40 cases with degenerative knee joint disease. *China Health News* 2014;8(19):136-36.

216. D Yang. Warm and regular acupuncture treatment of degenerative knee osteoarthritis *Public health* 2014;02(3):47.

217. B Yao, W Lei, B Yao, et al. Muscle strength training for elderly patients with knee osteoarthritis. *Chinese Journal of Rehabilitation* 2014;3(29):200-02.

218. W Zhou. Clinical Comparative Study of Electro-acupuncture and Acupuncture Debate Temperature on Kidney Marrow Deficiency Syndrome of Knee Osteoarthritis. *Journal of Emergency in Traditional Chinese Medicine* 2014;8(23):1521-23.

219. L Zhou. Clinical Observation of Moxibustion in Treatment of Knee Osteoarthritis. *Shanghai Journal of Acupuncture and Moxibustion* 2014;12(33):1086-88.

220. J Zhu, G Wang, X Jin, et al. Effect of Electroacupuncture on Serum MMP-3 in Senile Knee Osteoarthritis. *Chinese Archives of Traditional Chinese Medicine* 2014;2(32):363-64.

221. M Chen. Acupuncture combined with Fu's Jing-moxibustion treatment on knee osteoarthritis：randomized controlled trial [Doctor]. Guangzhou University of Chinese Medicine, 2015.

222. Y Chen. The scraping treatment for early knee osteoarthritis [Master]. Fujian University of Traditional Chinese Medicine, 2015.

223. X Feng, W Zhou, H Xu, et al. The muscle therapy for knee osteoarthritis *Massage and rehabilitation medicine* 2015;22(6):13-15.

224. J He. The Effect on Serum Cytokines and Knee Osteoarthritis by Acupuncture Combined Linggui bafa and Chinese Herbs [Doctor]. Guangzhou University of Chinese Medicine, 2015.

225. H Hong, T Wang, Y Xie, et al. Combination therapy of Shi’s acupuncture therapy and oral application of Yishen Juanbi Wan for the treatment of knee osteoarthritis. *The Journal of Traditional Chinese Orthopedics and Traumatology* 2015;5(27):5-8.

226. C Li. Clinical Observation of Seventy-six Cases of Keen Osteoarthritis Treated with Acupuncture and Manipulation. *Journal of Practical Traditional Chinese Internal Medicine* 2015;5(2):21-22.

227. S Li, Z Lin, P Lu, et al. Clinical research on treating knee osteoarthritis by small knife acupuncture. *Clinical Journal of Chinese Medicine* 2015;34(7):39-40.

228. X Li, H Wang. Treating 162 Cases of Knee Osteoarthritis by Needle-knife Lysis Based on Position Differentiation and Points Fixed. *Gansu Journal of Traditional Chinese Medicine* 2015;6(28):118-20.

229. Z Li, D Li. Electric acupuncture treatment on knee osteoarthritis: 38 cases. *Henan Traditional Chinese Medicine* 2015;2(35):300-02.

230. Z Li, J Wang, K Liu. An observation of the short-term therapeutic effect of deep kneading-pressing manipulation on the elderly knee osteoarthritis. *China Journal of Rehabilitation Medicine* 2015;6(30):580-84.

231. C Liang, J Cai, L Yan, et al. Needle knife therapy to improve the curative effect of knee osteoarthritis. *The Journal of Traditional Chinese Orthopedics and Traumatology* 2015;9(27):9-14.

232. Z Liang, Y Hu, Y Ding, et al. Clinical observation of needle knife for the treatment of knee osteoarthritis. *Chinese Journal of Traditional Medical Science and Technology* 2015;1(0):77-78.

233. W Lin, K Wu. Warm acupuncture in patients with rheumatoid arthritis of knee pain *Heilongjiang Medicine* 2015;2(28):376-77.

234. C Liu. Lost thorn with warm acupuncture treatment on knee osteoarthritis: 35 cases *Journal of Practical Traditional Chinese Medicine* 2015;1(31):53-54.

235. X Liu, Y Wang, C Li, et al. Influence of arthroscopy cleaning technique combining with electric acupuncture on cytokines and matrix metalloproteinases in synovial fluid of patients with knee osteoarthritis. *Journal of Hainan Medical College* 2015;12(21):1643-45.

236. X Lu. Clinical observation of warm acupuncture treatment of elderly knee osteoarthritis：81 cases *Chinese Journal of Ethnomedicine and Ethnopharmacy* 2015;8(24):62.

237. M Pan, Q Huang. Acupuncture point treatment on knee osteoarthritis. *Journal of Medicine ＆ Pharmacy of Chinese Minorities* 2015;7(21):3-5.

238. S Pan, J Huang, K Zhao, et al. Clinical Observation on Needling Combined with Ossotide Point Injection in Treatment of KOA with Liver-Kidney Deficiency. *Liaoning Journal of Traditional Chinese Medicine* 2015;1(42):154-57.

239. J Pei, R Mo, S Xu, et al. Needle knife release technique with massage in patients with knee osteoarthritis *Journal of Practical Traditional Chinese Medicine* 2015;8(31):760-61.

240. Y Sheng, Q Jia, G Shu, et al. Clinical study on treatment of knee osteoarthritis by acupuncture combined with ultrasonic. *Journal of Henan University of Chinese Medicine* 2015;8(30):

241. F Wang. Analysis of Treatment Effect of Warming Needle Moxibustion on Knee Osteoarthritis of Deficiency Cold Type. *Journal of Medical Research* 2015;10(2):164-66.

242. H Wang, X Li, C Zhang. Traditional Chinese medicine iontophoresis with functional exercise treatment on knee osteoarthritis *Journal of Practical Traditional Chinese Medicine* 2015;8(31):762.

243. L Wang, D Xu, H Ji, et al. Clinical Observation on the Treatment of Knee Osteoarthritis with Blade-needle. *RHEUNATISM AND ARTHRITIS* 2015;9(4):20-22.

244. W Wang, P Gen, D Chen, et al. Fire acupuncture treatment on knee osteoarthritis *Practical Clinical Journal of Integrated Traditional Chinese and Western Medicine* 2015;5(2):29-30,41.

245. Z Wang. Fire needle combined TCM internal treatment on knee osteoarthritis：39 cases. *Traditional Chinese Medicinal Research* 2015;2(28):45-47.

246. Y Chang, H Chang, Y Liu, et al. The effects of needle knife therapy on the C-reactive protein in patients with knee osteoarthritis. *Hebei Journal of Traditional Chinese Medicine* 2013;12(35):1846-47.

247. H Wu, J Liu, Y Zhang. Clinical choice of tactics of intervention on knee osteoarthritis. *Journal of Clinical Acupuncture and Moxibustion* 2012;9(28):35-36.

248. Z Zhen. Needle knife coordination technique treatment knee osseous arthritis clinical observation. *Massage and rehabilitation medicine* 2011;2(2):81-82.

249. L Ji. Clinical research on cytokine in serum of knee osteoarthritis patients with acupuncture treatment [Master]. Nanjing University of Traditional Chinese Medicine, 2012.

250. J Liu, X He, J Kan. Warm needle with rehabilitation training to improve the curative effect observation of knee osteoarthritis. Acupuncture and moxibustion societies academic essays Xichang, 2012:41-44.

251. H Wu. Warm acupuncture treatment on knee osteoarthritis *World Latest Medicine* 2015;34(15):104-04.

252. J Wu, F Li, Z Zhen, et al. Blade, fire needle combined isolation ginger moxibustion treatment on knee osteoarthritis. *World Latest Medicine* 2015;18(1):104-04.

253. W Wu. Electroacupuncture point treatment on knee osteoarthritis *Shanghai Journal of Traditional Chinese Medicine* 2015;6(49):63-65.

254. H Ying, F Huang, H Ying. Effect of Needle Warming Moxibustion and Electroacupuncture in Treatment of Knee Osteoarthritis. *Journal of Zhejiang University of Traditional Chinese Medicine* 2015;2(2):150-52.

255. S Zhang, J Liu. Fire needle plus moxibustion treatment on the arthritis of the knee. *Jiangxi Journal of Traditional Chinese Medicine* 2015;4(5):53-54,63.

256. H Zhao, L Ba. Temperature effects of acupuncture treatment of senile knee osteoarthritis research. *The Chinese and foreign women's health study* 2015;8(0):172-72.

257. R Zhong, H Cha, Z Fan, et al. Clinical Observations on Superficial Surround Needling plus Quadriceps Femoris Training for the Treatment of Knee Osteoarthritis. *Shanghai Journal of Acupuncture and Moxibustion* 2015;10(34):986-89.

258. T Zhou, Z Chen, Y Qian, et al. Clinical analysis of low-frequency electro-acupuncture for knee osteoarthritis. *Shanghai Journal of Traditional Chinese Medicine* 2015;8(49):56-57.

259. J Chang, T Jiang, J Yang, et al. Acupuncture and moxibustion treatment of knee osteoarthritis: 40 cases. *Yunnan Journal of Traditional Chinese Medicine and Materia Medica* 2016;03(2):52-53.

260. Y Guo, Y Liang, J Gu, et al. Treatment on early and middle stage knee osteoarthritis by electro surround acupuncture. *Jilin Journal of Traditional Chinese Medicine* 2016;1(0):91-93.

261. D Huang, H Gao, X Lai, et al. Electroacupuncture treatment on knee osteoarthritis *Journal of Shaanxi College of Traditional Chinese Medicine* 2016;02(7):66-68.

262. B Wang, X Liu, Z Hu, et al. YANG's pricking-cupping therapy for knee osteoarthritis：a multi-center randomized controlled trial. *Chinese Acupuncture ＆ Moxibustion* 2016;02(2):113-18.

263. W Wu, T He. Clinical efficacy analysis of fire needle treatment on knee osteoarthritis *Modern Diagnosis ＆ Treatment* 2016;01(4):38-40.

264. H Yang. Study on Knee Osteoarthritis Treated by Songjin Needle. *Asia-Pacific Traditional Medicine* 2016;2(12):97-99.

265. BV Christensen, IU Iuhl, H Vilbek, et al. Acupuncture treatment of severe knee osteoarthrosis. A long-term study. *Acta Anaesthesiol Scand* 1992;6(36):519-25.

266. W Takeda, J Wessel. Acupuncture for the treatment of pain of osteoarthritic knees. *Arthritis Care Res* 1994;3(7):118-22.

267. BM Berman, BB Singh, L Lao, et al. A randomized trial of acupuncture as an adjunctive therapy in osteoarthritis of the knee. *Rheumatology (Oxford)* 1999;4(38):346-54.

268. M Yurtkuran, T Kocagil. TENS, electroacupuncture and ice massage: comparison of treatment for osteoarthritis of the knee. *Am J Acupunct* 1999;3-4(27):133-40.

269. C Sangdee, S Teekachunhatean, K Sananpanich, et al. Electroacupuncture versus diclofenac in symptomatic treatment of osteoarthritis of the knee: a randomized controlled trial. *BMC Complement Altern Med* 2002;2):3.

270. MM Ng, MC Leung, DM Poon. The effects of electro-acupuncture and transcutaneous electrical nerve stimulation on patients with painful osteoarthritic knees: a randomized controlled trial with follow-up evaluation. *J Altern Complement Med* 2003;5(9):641-9.

271. BM Berman, L Lao, P Langenberg, et al. Effectiveness of acupuncture as adjunctive therapy in osteoarthritis of the knee: a randomized, controlled trial. *Ann Intern Med* 2004;12(141):901-10.

272. E Tukmachi, R Jubb, E Dempsey, et al. The effect of acupuncture on the symptoms of knee osteoarthritis--an open randomised controlled study. *Acupunct Med* 2004;1(22):14-22.

273. J Vas, C Mendez, E Perea-Milla, et al. Acupuncture as a complementary therapy to the pharmacological treatment of osteoarthritis of the knee: randomised controlled trial. *Bmj* 2004;7476(329):1216.

274. C Witt, B Brinkhaus, S Jena, et al. Acupuncture in patients with osteoarthritis of the knee: a randomised trial. *Lancet* 2005;9480(366):136-43.

275. HP Scharf, U Mansmann, K Streitberger, et al. Acupuncture and knee osteoarthritis: a three-armed randomized trial. *Ann Intern Med* 2006;1(145):12-20.

276. CM Witt, S Jena, B Brinkhaus, et al. Acupuncture in patients with osteoarthritis of the knee or hip: a randomized, controlled trial with an additional nonrandomized arm. *Arthritis Rheum* 2006;11(54):3485-93.

277. NE Foster, E Thomas, P Barlas, et al. Acupuncture as an adjunct to exercise based physiotherapy for osteoarthritis of the knee: randomised controlled trial. *Bmj* 2007;7617(335):436.

278. RC Tsang, PL Tsang, CY Ko, et al. Effects of acupuncture and sham acupuncture in addition to physiotherapy in patients undergoing bilateral total knee arthroplasty--a randomized controlled trial. *Clin Rehabil* 2007;8(21):719-28.

279. L Williamson, MR Wyatt, K Yein, et al. Severe knee osteoarthritis: a randomized controlled trial of acupuncture, physiotherapy (supervised exercise) and standard management for patients awaiting knee replacement. *Rheumatology (Oxford)* 2007;9(46):1445-9.

280. M Yurtkuran, A Alp, S Konur, et al. Laser acupuncture in knee osteoarthritis: a double-blind, randomized controlled study. *Photomed Laser Surg* 2007;1(25):14-20.

281. K Itoh, S Hirota, Y Katsumi, et al. A pilot study on using acupuncture and transcutaneous electrical nerve stimulation (TENS) to treat knee osteoarthritis (OA). *Chin Med* 2008;3):2.

282. K Itoh, S Hirota, Y Katsumi, et al. Trigger point acupuncture for treatment of knee osteoarthritis--a preliminary RCT for a pragmatic trial. *Acupunct Med* 2008;1(26):17-26.

283. RW Jubb, ES Tukmachi, PW Jones, et al. A blinded randomised trial of acupuncture (manual and electroacupuncture) compared with a non-penetrating sham for the symptoms of osteoarthritis of the knee. *Acupunct Med* 2008;2(26):69-78.

284. H Lansdown, K Howard, S Brealey, et al. Acupuncture for pain and osteoarthritis of the knee: a pilot study for an open parallel-arm randomised controlled trial. *BMC Musculoskelet Disord* 2009;10):130.

285. Y WU, J Zhang, S Li. Clinical Observation on Treatment of Knee Osteoarthritis by Warm Needling Method. *Acupuncture and massage* 2009;6(5):349-51.

286. ME Suarez-Almazor, C Looney, Y Liu, et al. A randomized controlled trial of acupuncture for osteoarthritis of the knee: effects of patient-provider communication. *Arthritis Care Res (Hoboken)* 2010;9(62):1229-36.

287. P White, P Prescott, G Lewith. Does needling sensation (de qi) affect treatment outcome in pain? Analysis of data from a larger single-blind, randomised controlled trial. *Acupunct Med* 2010;3(28):120-5.

288. S Lev-Ari, E Miller, Y Maimon, et al. Delayed effect of acupuncture treatment in OA of the knee: A blinded, randomized, controlled trial. *Evidence-based Complementary and Alternative Medicine* 2011;792975(2011 (no pagination)):

289. CI Mavrommatis, E Argyra, A Vadalouka, et al. Acupuncture as an adjunctive therapy to pharmacological treatment in patients with chronic pain due to osteoarthritis of the knee: a 3-armed, randomized, placebo-controlled trial. *Pain* 2012;8(153):1720-6.

290. LX Chen, JJ Mao, S Fernandes, et al. Integrating acupuncture with exercise-based physical therapy for knee osteoarthritis: a randomized controlled trial. *J Clin Rheumatol* 2013;6(19):308-16.

291. M Saleki, T Ahadi, M Razi, et al. Comparison of the effects of acupuncture and isometric exercises on symptom of knee osteoarthritis. *Int J Prev Med* 2013;Suppl 1(4):S73-7.

292. A Ashraf, F Zarei, MJ Hadianfard, et al. Comparison the effect of lateral wedge insole and acupuncture in medial compartment knee osteoarthritis: a randomized controlled trial. *Knee* 2014;2(21):439-44.

293. X Chen, RB Spaeth, K Retzepi, et al. Acupuncture modulates cortical thickness and functional connectivity in knee osteoarthritis patients. *Sci Rep* 2014;4):6482.

294. RS Hinman, P McCrory, M Pirotta, et al. Acupuncture for chronic knee pain: a randomized clinical trial. *Jama* 2014;13(312):1313-22.

295. M Lin, X Li, W Liang, et al. Needle-knife therapy improves the clinical symptoms of knee osteoarthritis by inhibiting the expression of inflammatory cytokines. *Exp Ther Med* 2014;4(7):835-42.

296. H Qu, R Qu, J Liu, et al. Clinical observation on warm needling in canicular days for knee osteoarthritis. *Journal of Acupuncture and Tuina Science* 2015;01(13):63-66.

297. Zhang Y, Bao F, Wang Y, et al. Influence of acupuncture in treatment of knee osteoarthritis and cartilage repairing. *Am J Transl Res* 2016;9:3995-4002.

298. White A, Tough L, Eyre V, et al. Western medical acupuncture in a group setting for knee osteoarthritis: results of a pilot randomised controlled trial. *Pilot Feasibility Stud* 2016;10.

299. Helianthi DR, Simadibrata C, Srilestari A, et al. Pain Reduction After Laser Acupuncture Treatment in Geriatric Patients with Knee Osteoarthritis: a Randomized Controlled Trial. *Acta Med Indones* 2016;2:114-21.

300. Chen X, Spaeth RB, Freeman SG, et al. The modulation effect of longitudinal acupuncture on resting state functional connectivity in knee osteoarthritis patients. *Mol Pain* 2015;67.

301. Plaster R, Vieira WB, Alencar FA, et al. Immediate effects of electroacupuncture and manual acupuncture on pain, mobility and muscle strength in patients with knee osteoarthritis: a randomised controlled trial. *Acupunct Med* 2014;3:236-41.

302. Martins F, Kaster T, Schutzler L, et al. Factors influencing further acupuncture usage and a more positive outcome in patients with osteoarthritis of the knee and the hip: a 3-year follow-up of a randomized pragmatic trial. *Clin J Pain* 2014;11:953-9.

303. Al Rashoud AS, Abboud RJ, Wang W, et al. Efficacy of low-level laser therapy applied at acupuncture points in knee osteoarthritis: a randomised double-blind comparative trial. *Physiotherapy* 2014;3:242-8.

304. Spaeth RB, Camhi S, Hashmi JA, et al. A longitudinal study of the reliability of acupuncture deqi sensations in knee osteoarthritis. *Evid Based Complement Alternat Med* 2013;204259.

305. Karner M, Brazkiewicz F, Remppis A, et al. Objectifying specific and nonspecific effects of acupuncture: a double-blinded randomised trial in osteoarthritis of the knee. *Evid Based Complement Alternat Med* 2013;427265.

306. Lu TW, Wei IP, Liu YH, et al. Immediate effects of acupuncture on gait patterns in patients with knee osteoarthritis. *Chin Med J (Engl)* 2010;2:165-72.

307. Huang W, Bliwise DL, Carnevale CV, et al. Acupuncture for pain and sleep in knee osteoarthritis. *J Am Geriatr Soc* 2010;6:1218-20.

308. Taechaarpornkul W, Suvapan D, Theppanom C, et al. Comparison of the effectiveness of six and two acupuncture point regimens in osteoarthritis of the knee: a randomised trial. *Acupunct Med* 2009;1:3-8.

309. Shen X, Zhao L, Ding G, et al. Effect of combined laser acupuncture on knee osteoarthritis: a pilot study. *Lasers Med Sci* 2009;2:129-36.

310. Meng CR, Fan L, Fu WB, et al. Clinical research on abdominal acupuncture plus conventional acupuncture for knee osteoarthritis. *J Tradit Chin Med* 2009;4:249-52.

311. Ahsin S, Saleem S, Bhatti AM, et al. Clinical and endocrinological changes after electro-acupuncture treatment in patients with osteoarthritis of the knee. *Pain* 2009;1-3:60-6.

312. Brinkhaus B, Witt CM, Jena S, et al. Physician and treatment characteristics in a randomised multicentre trial of acupuncture in patients with osteoarthritis of the knee. *Complement Ther Med* 2007;3:180-9.

313. Tillu A, Roberts C, Tillu S. Unilateral versus bilateral acupuncture on knee function in advanced osteoarthritis of the knee--a prospective randomised trial. *Acupunct Med* 2001;1:15-8.

314. Singh BB, Berman BM, Hadhazy V, et al. Clinical decisions in the use of acupuncture as an adjunctive therapy for osteoarthritis of the knee. *Altern Ther Health Med* 2001;4:58-65.

315. Kwon YB, Kim JH, Yoon JH, et al. The analgesic efficacy of bee venom acupuncture for knee osteoarthritis: a comparative study with needle acupuncture. *The American journal of Chinese medicine* 2001;2:187-99.

316. Berman BM, Lao L, Greene M, et al. Efficacy of traditional Chinese acupuncture in the treatment of symptomatic knee osteoarthritis: a pilot study. *Osteoarthritis Cartilage* 1995;2:139-42.

317. Huang J, Zhang W, Lou BD, et al. Observation on clinical efficacy of tension-balance acupuncture therapy in treating knee osteoarthritis. *World Journal of Acupuncture - Moxibustion* 2014;2:42-46.

318. Fu MY, Zhang ZL. Knee osteoarthritis treated with acupuncture based on syndrome differentiation: A randomized controlled trial. *World Journal of Acupuncture - Moxibustion* 2012;3:11-17.
