# Supplementary material for: The quality of reporting in randomized controlled trials of acupuncture for knee osteoarthritis: A cross-sectional survey
Source: PLoS One. 2018 Apr 12;13(4):e0195652. doi: 10.1371/journal.pone.0195652 (PMC5896985; doi:10.1371/journal.pone.0195652)
Supplement: S4 File — (DOCX) [file pone.0195652.s004.docx]

**Sensitivity analyses**

**Table 1: The total score and the CONSORT score using the alternative approach under sensitivity analysis**

| **Summarized scores** | **Total**  **（n=318, %）** | **Chinese**  **(n=264, %)** | **English**  **(n=54, %)** |
| --- | --- | --- | --- |
| Standard CONSORT^‡^ | 15 (13-17) | 15 (13-17) | 18(15-26) |
| CONSORT Extension^‡^ | 9 (9-9) | 9 (9-9) | 4 (3-5) |
| STRICTA**^‡^** | 9 (8-10) | 9 (7-10) | 9 (7-11) |
| Total scores **^a‡^** | 33 (30-36) | 32 (30-35) | 37 (33-45) |

**^a^:** The score was calculate based on the items of the standard CONSORT, CONSORT Extension and STRICTA checklist **^‡:^** Score was showed as Median (interquartile range)

**Table 2: Factors associated with overall reporting quality^a^**

| **Variables** | **Univariable analysis** | | ***P*** | **Multivariable analysis** | | ***P*** |
| --- | --- | --- | --- | --- | --- | --- |
|  | **Coefficient ( 95% CI)** | |  | **Coefficient ( 95% CI)** | |  |
| **The main effect of the primary outcome** |  | | | | | |
| significant vs non-significant_[ref]_ | 1.78 (-0.78 to 2.50) | 0.45 | | -0.09 (-3.24 to 2.07) | | 0.88 |
| **Language** |  |  | |  |  |  |
| English vs Chinese_[ref]_ | 12.45 (7.34 to 15.73) | <0.001 | | 9.56 (7.65 to 10.44) | | <0.001 |
| **Author’s affiliation to statistics or epidemiology department** |  |  | |  |  |  |
| yes versus no_[ref]_ | 13.25 (10.37 to 16.23) | <0.001 | | 9.45 (5.55 to 14.24) | | <0.001 |
| **Center** |  |  | |  |  |  |
| multicenter vs single center_[ref]_ | 9.65 (6.53 to 16.24) | <0.001 | | 5.48 (2.87 to 7.66) | | <0.001 |
| **Sample size** |  |  | |  | |  |
| >80 vs ≤80_[ref]_ | 0.53 (-3.24 to 2.00) | 0.49 | | 0.43 (-0.77 to 1.57) | | 0.67 |

Note: [ref]: reference level

^a^: as indicated by the total score calculated based on the items of the standard CONSORT, CONSORT

Extension for Trials Assessing Non-Pharmacological Treatments and STRICTA checklist

**Table 3: Factors associated with the standard CONSORT**

| **Variables** | **Univariable analysis** | ***P*** | **Multivariable analysis** | | ***P*** |
| --- | --- | --- | --- | --- | --- |
|  | **Coefficient (95% CI)** |  | **Coefficient (95% CI)** | |  |
| **The main effect of the primary outcome** |  | | | | |
| significant vs non-significant_[ref]_ | 0.74 (-0.35 to 2.17) | 0.34 | 0.23 (-0.64 to 1.02) | | 0.34 |
| **Language** |  |  |  |  |  |
| English vs Chinese_[ref]_ | 7.55 (6.24 to 8.33) | <0.001 | 4.33 (2.37 to 7.64) | | <0.001 |
| **Author’s affiliation to statistics or epidemiology department** |  |  |  |  |  |
| yes versus no_[ref]_ | 9.53 (7.56 to 13.21) | <0.001 | 6.00 (2.33 to 8.45) | | <0.001 |
| **Center** |  |  |  |  |  |
| multicenter vs single center_[ref]_ | 4.72 (2.89 to 8.34) | <0.001 | 2.67 (1.16 to 5.03) | | <0.001 |
| **Sample size** |  |  |  | |  |
| >80 vs ≤80_[ref]_ | 0.18 (-0.46 to 1.78) | 0.48 | 0.007 (-0.84 to 0.66) | | 0.75 |

Note: _[ref]:_ reference level
