# Supplementary material for: The quality of reporting in randomized controlled trials of acupuncture for knee osteoarthritis: A cross-sectional survey
Source: PLoS One. 2018 Apr 12;13(4):e0195652. doi: 10.1371/journal.pone.0195652 (PMC5896985; doi:10.1371/journal.pone.0195652)
Supplement: S1 Table — (DOCX) [file pone.0195652.s005.docx]

**The evaluation checklist**

| **The standard CONSORT and the CONSORT Extension for Trials Assessing Non-Pharmacological Treatments** |
| --- |
| 1.Identification as a randomized trial in the title |
| 2.Structured summary of trial design, methods, results, and conclusions |
| 3.Description of the experimental treatment, comparator, care providers, centers and blinding status**^†^** |
| 4.Scientific background and explanation of rationale |
| 5.Specific objectives or hypotheses |
| 6.Description of ① trial design (such as parallel, factorial) including ② allocation ratio  We rated item 6 as ‘yes’ only if one of the two subitems was reported. |
| 7.①Important changes to methods after trial commencement (such as eligibility criteria), ②with reasons.  We rated item 7 as ‘yes’ only if one of the two subitems was reported |
| 8.Eligibility criteria for participants  9.When applicable, eligibility criteria for centers and those performing the interventions^a^**^†^** |
| 10.① Settings and ② locations where the data were collected  We rated item 10 as ‘yes’ only if one of the two subitems was reported |
| 11.Completely ①defined pre-specified primary and secondary outcome measures, including② how and when they were assessed  We rated item 11 as ‘yes’ only if one of the two subitems was reported |
| 12.①Any changes to trial outcomes after the trial commenced, ②with reasons  We rated item 12 as ‘yes’ only if one of the two subitems was reported |
| 13.How sample size was determined 14.When applicable, details of whether and how the clustering by care providers or centers was addressed^a^**^†^** |
| 15.When applicable, explanation of any① interim analyses and ②stopping guidelines^a^  We rated item 15 as ‘yes’ only if one of the two subitems was reported |
| 16.Method used to generate the random allocation sequence  17.When applicable, how care providers were allocated to each trial group^a^**^†^** |
| 18.①Type of randomization; ②details of any restriction (such as blocking and block size)  We rated item 18 as ‘yes’ only if one of the two subitems was reported |
| 19.Mechanism used to implement the random allocation sequence (such as sequentially numbered containers), describing any steps taken to conceal the sequence until interventions were assigned |
| 20.①Who generated the random allocation sequence, ② who enrolled participants, and ③who assigned participants to interventions  We rated item 20 as ‘yes’ only if one of the three subitems was reported |
| 21.If done, ①who was blinded after assignment to interventions (for example, participants, care providers, those assessing outcomes) and②how^a^  We rated item 21 as ‘yes’ only if the outcome assessor was blinded |
| 22.Whether or not those administering co-interventions were blinded to group assignment^a^**^†^**  23.If relevant, description of the similarity of interventions^a^ |
| 24.Statistical methods used to compare groups for primary and secondary outcomes  We rated item 24 as ‘yes’ if the author specifies the statistical methods for all outcome or primary outcome. |
| 25.When applicable, details of whether and how the clustering by care providers or centers was addressed^a^**^†^**  26.Methods for additional analyses, such as subgroup analyses and adjusted analyses |
| 27.For each group, ① the numbers of participants who were randomly assigned, ② received intended treatment, and were analysed for the primary outcome  We rated item 27 as ‘yes’ only if one of the two subitems was reported |
| 28.The number of care providers or centers performing the intervention in each group and the number of patients treated by each care provider or in each center**^†^**  29. For each group, ①losses and exclusions after randomisation, ② together with reasons  We rated item 29 as ‘yes’ only if one of the two subitems was reported |
| 30.Dates defining the periods of recruitment and follow-up |
| 31.Why the trial ended or was stopped |
| 32.A table showing baseline demographic and clinical characteristics for each group  33.When applicable, a description of care providers (case volume, qualification, expertise, etc.) and centers (volume) in each group^a^**^†^** |
| 34.For each group, number of participants (denominator) included in each analysis and whether the analysis was by original assigned groups |
| 35.For each primary and secondary outcome, results for each group, and the estimated effect size and its precision (such as 95% confidence interval);  36.For binary outcomes, presentation of both absolute and relative effect sizes is recommended^a^ |
|  |
| 37.Results of any other analyses performed, including subgroup analyses and adjusted analyses, distinguishing pre-specified from exploratory |
| 38.All important harms or unintended effects in each group |
| 39.Trial limitations, addressing sources of potential bias, imprecision, and, if relevant, multiplicity of analyses |
| 40.Generalizability (external validity, applicability) of the trial findings  41.Generalizability (external validity) of the trial findings according to the intervention, comparators, patients, and care providers and centers involved in the trial**^†^** |
| 42.Interpretation consistent with results, balancing benefits and harms, and considering other relevant evidence |
| 43.Take into account the choice of the comparator, lack of or partial blinding, unequal expertise of care providers or centers in each group**^†^**  44.Registration number and name of trial registry |
| 45.Where the full trial protocol can be accessed, if available |
| 46.Sources of funding and other support (such as supply of drugs), role of funders |
| **STRICTA checklist** |
| 47.Style of acupuncture (e.g., Traditional Chinese Medicine, Japanese, Korean, Western medical, Five Element, ear acupuncture, etc.) |
| 48.Reason for the treatment provided, based on the historical context, literature sources and/or consensus methods, with references where appropriate |
| 49.Extent to which treatment was varied |
| 50.Number of needle insertions per subject per session (the mean and range where relevant) |
| 51.Names (or location if no standard name) of the points used (uni-/bilateral) |
| 52.Depth of insertion, based on a specified unit of measurement or on a particular tissue level |
| 53.Responses sought (e.g., de qi or muscle twitch response) |
| 54.Needle stimulation (e.g., manual or electrical) |
| 55.Needle retention time |
| 56.Needle type (diameter, length and manufacturer or material) |
| 57.Number of treatment sessions |
| 58.Frequency and duration of treatment sessions |
| 59.Details of other interventions administered to the acupuncture group (e.g., moxibustion, cupping, herbs, exercises, lifestyle advice) |
| 60.Setting and context of treatment, including instructions to practitioners, and information and explanations to patients |
| 61.Description of participating acupuncturists (qualification or professional affiliation, years in acupuncture practice, other relevant experience) |
| 62.Rationale for the control or comparator in the context of the research question, with sources that justify the choice(s) |
| 63.Precise description of the control or comparator. If sham acupuncture or any other type of acupuncture-like control was used, provided details as for items 50–59 above |

| **^†^**: Items related to CONSORT Extension for Trials Assessing Non-Pharmacological Treatments  ^a^: Items with the situation of ‘when applicable’, ‘if done’, or ‘if relevant’ |
| --- |
